# Bioinspired spiking architecture enables energy constrained touch encoding

**DOI:** 10.1038/s41467-026-68858-7

**Published:** 2026-01-28

**Authors:** Andrea Ortone, Mariangela Filosa, Giacomo Indiveri, Giuseppe Desoli, Alberto Mazzoni, Calogero Maria Oddo

**Affiliations:** 1 The BioRobotics Institute, Sant’Anna School of Advanced Studies, Pisa, Italy; 2 Department of Excellence in Robotics and AI, Sant’Anna School of Advanced Studies, Pisa, Italy; 3 Interdisciplinary Research Center Health Science, Sant’Anna School of Advanced Studies, Pisa, Italy; 4 Institute of Neuroinformatics, University of Zurich and ETH Zurich, Zurich, Switzerland; 5 STMicroelectronics, Cornaredo, Italy

**Keywords:** Touch receptors, Biomedical engineering, Electrical and electronic engineering, Neural circuits

## Abstract

The sense of touch is essential for safe interactions with the external world, enabling humans to rapidly detect and localize physical stimuli. Biological systems achieve these abilities through hundreds of thousands of mechanoreceptors distributed across the skin and efficiently processing vast streams of tactile information. Replicating these affordances in autonomous systems is crucial for advancing robotics. However, current tactile sensing solutions face critical challenges, including excessive wiring, high energy demnds of AI computing, and limitations in scalability and parallel processing. Here, we present a modular artificial tactile system combining a Fiber Bragg Grating-based e-skin with a spiking neural network (SNN) that mimics the early stages of the human somatosensory system. Our architecture achieves up to 10× localization super-resolution, improving localization accuracy by 32% over state-of-the-art deep learning methods and effectively generalizing to multitouch and dynamic conditions. Crucially, when implemented on a neuromorphic chip, the SNN demonstrates robustness to the constrained resolution and mismatches of analog neurons, bolstering highly parallel and sub-mWatt hardwired computation. Bioinspired connectivity is shown to functionally influence tactile processing, offering mechanistic insights in a framework that bridges physiological hypotheses, modeling, and validation in a real-world tactile scenario. These results demonstrate a scalable, energetically sustainable solution for touch perception, with immediate applications in autonomous systems requiring safe human interaction and operation in dynamic environments.

## Abstract (web summary)

Current tactile sensing solutions face challenges in excessive wiring, high energy demands of AI computing, and limitations in scalability and parallel processing. Here, the authors present a neuromorphic tactile system combining artificial skin and spiking hardware encodes touch with high accuracy at low power.

## Introduction

The ability to sense and process tactile stimuli is crucial for safe interaction in dynamic and unpredictable environments, enabling humans to explore and navigate their surroundings^1^ and to develop and leverage a sense of embodiment^2^. To pursue these tasks, the sense of touch has evolved into a complex sensory modality^3,4^, integrating complementary mechanoreceptors^5,6^ that transduce and encode large amounts of spatio-temporal information^7^. Unlike other senses, tactile perception is distributed since it conveys stimuli from the skin, covering the whole body. This widespread distribution bolsters the necessity for the tactile sensory system to adopt energy-efficient computing strategies^8^. Reproducing these features in artificial tactile systems, making them biomimetic^9,10^ would be pivotal to enable significant progress in robotic^11,12^ and biomedical applications^13,14^.

Indeed, robots of the Industry 4.0 and 5.0 era are expected to physically interact with humans and autonomously navigate the external world^15^. To accomplish these tasks while ensuring safety, state of the art solutions often rely on localized sensing strategies. These include force/torque sensors^16^ or contactless proximity detectors^16,17^, which trade effectiveness in specific settings with a reduction in robot modularity and scalability. Promising advances in the design of large-area, modular and flexible electronic skins enhanced the possibility of distributing tactile perception across large portions of robot bodies^18–22^. As a result, the volume of data provided by tactile sensors has significantly increased, thereby bolstering the demand for effective integration and decoding of tactile information. In this regard, standard artificial deep neural networks have been proposed^23–28^, but often employed frame-based approaches, leading to limitations in latency, sensitivity to temporal information and energy consumption^28^. More recently, event-based solutions built on spiking neural networks^29^ (SNN) showed promising results in dynamic object classification through touch^30^ and pattern and textures classification^31–34^, demonstrating that spatiotemporal information can effectively be decoded through SNNs^35,36^. Yet, replicating the degree of robustness, scalability, and efficiency of biological somatosensory systems remains challenging, with key obstacles including the lack of end-to-end solutions for processing spatio-temporal information on scalable e-skins, with minimal power consumption, and reduced wiring complexity.

In this study, we present a bioinspired processing system encoding tactile information with constrained energy budget, enabling the detection and localization of stimuli applied on a large-area soft e-skin. The e-skin integrates Fiber Bragg Grating^37^ (FBG) sensors, strategically embedded in the silicon to capture spatio-temporal information of the applied stimuli, mimicking the response of slowly adapting primary afferents^5,6^. The FBG signals are processed by a fully event-driven architecture built on a SNN. Besides providing a faithful emulation of the early processing of the tactile system^38^, the proposed spike-based communication and computational approach effectively decode contact positions of applied stimuli and enhance power efficiency during both active and resting conditions. The SNN architecture is compatible with mixed-signal neuromorphic processors and has been successfully implemented on a DYNAP-SE chip^39^. In this setup, the tactile system exhibited strong resilience to the limited resolution and device mismatches typical of such technologies^40^, maintaining high decoding performance. While prior neuromorphic approaches have often emphasized and relied on deterministic implementations and spike-timing precision, the present work explores a complementary regime, which aligns with the intrinsically fluctuating and heterogeneous dynamics of analog circuits to enable robust, low-power and low-latency processing via SNNs. This approach supports distributed computations on digital-analog processors, which represent a valuable perspective for future energy-efficient AI solutions^41,42^.

Complementing these system-level contributions, we systematically explore the functional impact of alternative inhibitory connectivity motifs in biologically inspired networks. This comparative analysis, focused on feedforward, feedback, and hybrid inhibition schemes, is situated within a broader science-oriented methodological framework that bridges physiological hypotheses, computational modeling, and experimental validation in a real-world tactile scenario. By leveraging this convergence, our work offers mechanistic insights into early somatosensory processing and provides a quantitative perspective on why specific connectivity patterns may have been favored in biological systems for decoding tactile qualia.

Through a balanced trade-off between bio-emulative and engineering approaches, the presented solution establishes a robust and energy-efficient framework for tactile perception, that can be directly transferred to robotic and biomedical applications.

## Results

### Design of the neuromorphic tactile system

**Fig. 1:**
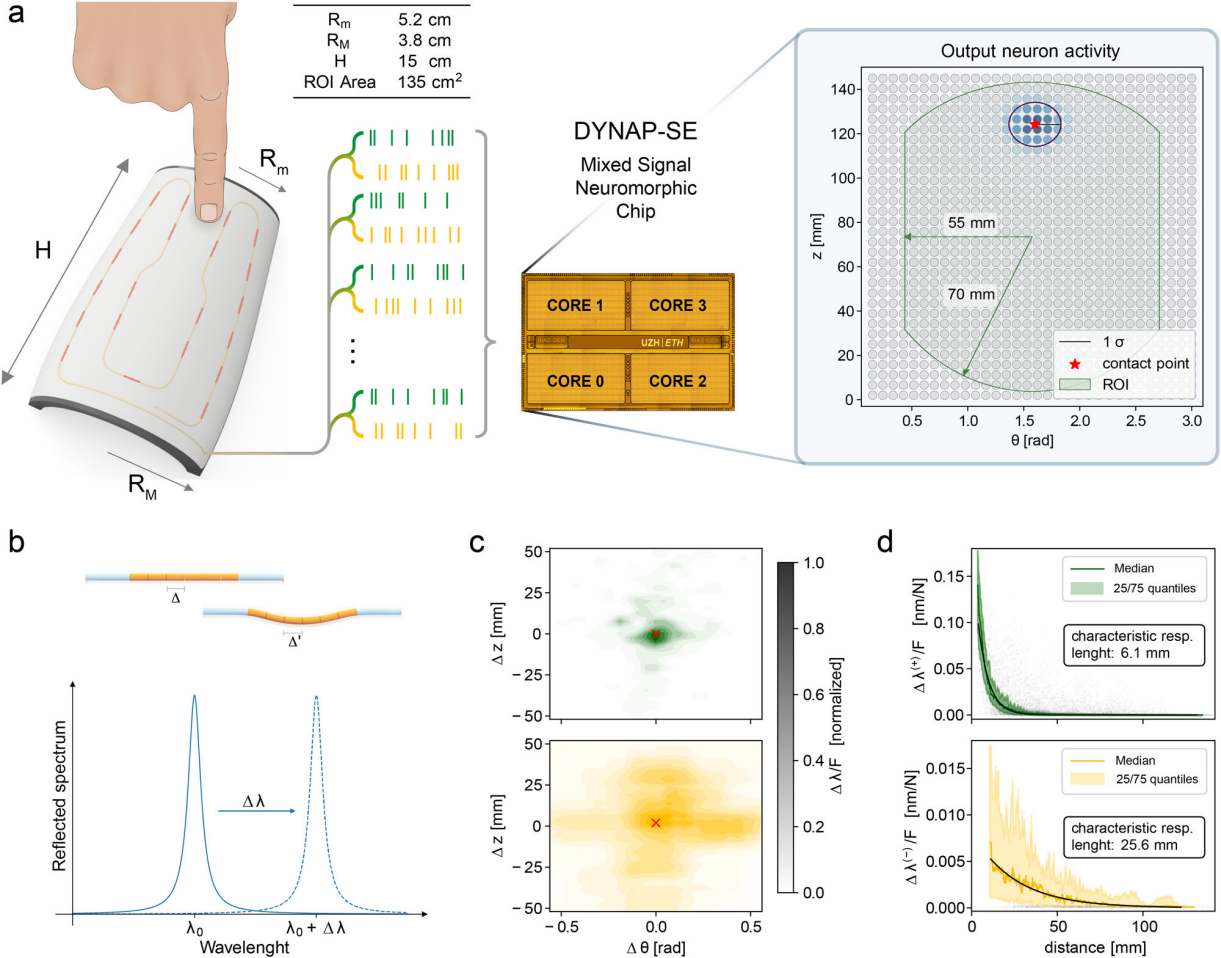
Representation of the e-skin and of the neuromorphic processing of tactile stimuli. **a** Design of the proposed system: (left) e-skin embedded with FBG sensors and spiking representations of the tactile signals; (right) die photo of the DYNAP-SE neuromorphic processor and activity of the output neurons of the spiking neural network when a punctual indentation is performed on the region of interest (ROI); **b** working principle of FBG transducers: local strain variations cause a shift (Δλ) in the reflected wavelength of each sensor; **c**, **d** receptive field (**c**) and radial sensitivity (**d**) of the positive (green) and negative (yellow) components of Δλ, mimicking SA1 and SA2 mechanoreceptors respectively.

The developed bioinspired tactile system comprises two main components to detect and localize tactile stimuli performed over large areas: an artificial skin integrating FBG sensors, and an online processing unit based on a SNN, implemented on a neuromorphic electronic chip^39^ as a low-power demonstrator (Fig. 1a).

The soft and modular e-skin^23^ (see Methods) integrates 21 FBG transducers beneath its 135 cm^2^ Region of Interest (ROI). Each FBG selectively reflects a wavelength of the injected light spectrum, which shifts proportionally to local strains (see Methods). Therefore, the magnitude of these shifts (Δλ_i_) encodes the intensity of the applied stimuli and the combination of this information from multiple sensors enables their localization (Fig. 1b). Notably, all transducers are inscribed into a single optical fiber and transmit their back-reflected spectra in parallel. This reduces the necessary wiring within the proposed system. The positioning of the FBG sensors within the e-skin has been engineered such that their responses to the applied stimuli functionally mimic the properties of slowly adapting mechanoreceptors^3,4^. The positive component of each sensor Δλ is sharply peaked (Fig. 1c) and features a rotationally symmetric receptive field $({\mathbb{E}}[{CV}]=1.1\pm 0.1)$ with a characteristic radius of 6.1 ± 0.1 mm (Fig. 1d), hence resembling the sensitivity to local stimuli of SA1 mechanoreceptors. Conversely, the receptive fields of the negative component of Δλ are broader (characteristic radius of 25.6 ± 0.8 mm) and less regular (${\mathbb{E}}$[CV] = 5.4 ± 0.3), which are consistent with a higher sensitivity to distributed transversal deformations caused by the poissonian response of the skin^23^, and functionally mimic the sensitivity of SA2 mechanoreceptors^2,3^ (see Fig. 1c, d). As in biological skin^5^, the receptive fields of the different sensors are overlapped (see Supplementary Fig. S4a), enabling the encoding of finer spatial information^43^.

Both components of the FBG sensor signals are pre-processed through a logarithmic transformation (see Methods), fed as external current to a first feedforward layer of Leaky Integrate and Fire neurons (LIF - see Methods) mimicking mechanoreceptors, and thereby converted into spike trains for further processing. This spike-based approach naturally performs data reduction in both active and resting conditions and fosters power efficiency^44^.

**Fig. 2:**
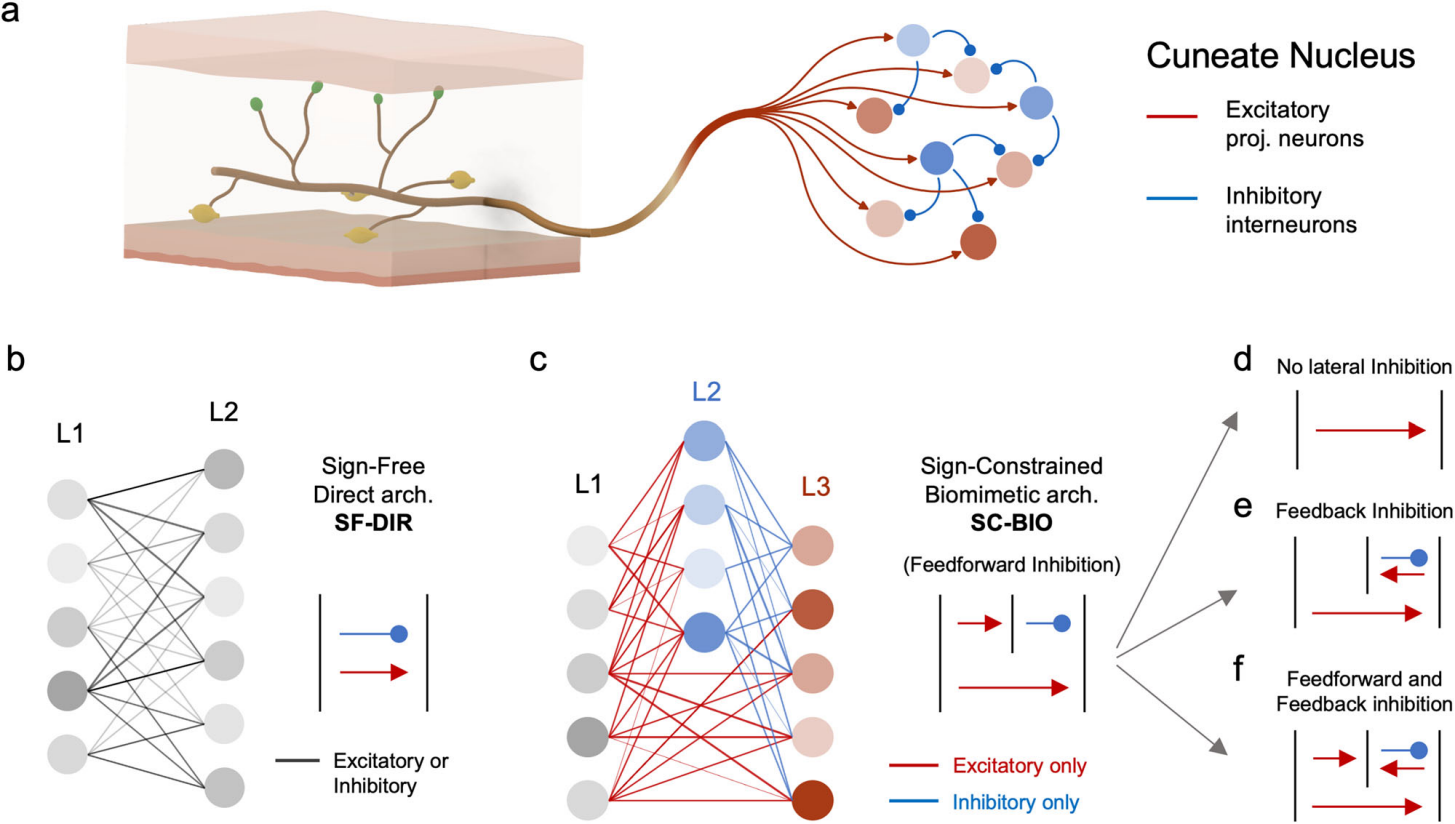
Representation of the considered neuronal architectures. **a** Connectivity between primary tactile afferents and neural populations in the Cuneate Nucleus; **b**, **c** schematic representations of the proposed architectures for the considered spiking neural networks. Lines between layers depict unidirectional structural connectivity, flowing from left to right. In the SF-DIR architecture (**b**), the mechanoreceptor layer (L1) directly connects to the output layer (L2), with connections that can dynamically switch between excitatory and inhibitory states during training. In the SC-BIO architecture (**c**), the structural connectivity mirrors that of the early somatosensory system (see subpanel a), with mechanoreceptor-activated primary sensory neurons (L1) being exclusively excitatory and interneurons (L2) purely inhibitory. **d–f** Schematic representations of three biologically plausible variants of the SC-BIO architecture**: d** suppression of lateral inhibition via removal of the interneuron population; **e** replacement of feedforward inhibition with feedback inhibition from the output layer to the population of interneurons, forming a recurrent loop; **f** combined architecture incorporating both feedforward and feedback inhibitory connections.

To investigate the optimality criteria of biologically inspired architectures, we implemented two main neuronal configurations in the following processing layers (see Fig. 2b, c). In the first architecture (SignFree DIRect, SF-DIR), a second layer receives feedforward direct inputs from the mechanoreceptor layer. In this configuration the connections are not constrained, i.e., they are allowed to switch from positive (i.e., excitatory) to negative (i.e., inhibitory) values during learning, a mechanism not implemented in biological neural networks. The second architecture (SignConstrained BIOinspired, SC-BIO), instead, is inspired by the properties and structural connectivity of the early somatosensory system^4,38,45^. In this configuration, neurons in the mechanoreceptor layer are excitatory and therefore establish only positive connections targeting both the neurons in the output layer (mimicking localization sensitive projection neurons in the Cuneate Nucleus^4^) and a population of interneurons. These latter interneurons are inhibitory, and form only negative connections directed to the output layer. This structure implements lateral inhibition via a feedforward inhibitory pathway, wherein excitatory mechanoreceptor-mimicking neurons activate inhibitory interneurons, which then inhibit the output neurons. This organization reflects recent experimental findings suggesting that inhibitory interneurons in the Dorsal Column Nuclei are predominantly driven by direct dorsal column afferents rather than by recurrent circuits^4^.

To further investigate the functional relevance of lateral inhibition and explore alternative bio-compatible connectivity schemes, we implemented three additional architectures. The first variant excludes lateral inhibition entirely by removing the interneuron population (see Fig. 2d). The second replaces feedforward inhibition with feedback inhibition, in which localization-sensitive output neurons project inhibitory connections to the interneurons, creating a recurrent circuit (see Fig. 2e). The third architecture combines feedforward and feedback inhibition, preserving the original feedforward structure and integrating feedback projections from the output neurons to the inhibitory interneurons (see Fig. 2f). In all considered networks, the intensity of the connections between the different layers and the individual baseline currents of each neuron are learned through training (see Methods). The dataset employed to train and evaluate the localization performance of the networks consists of 2026 indentations randomly applied over the surface of the e-skin and collected through an automated protocol (see Methods).

The output neurons of the two networks set up a somatotopic bidimensional map of the surface of the e-skin where each neuron *i* is associated with a given position (*θ*_*i*_*, z*_*i*_) and is trained such that its target activity *y*_i_^*^(t) reproduces a gaussian kernel around the actual stimulus position (see Eq. (12) in Methods). The positions associated with output neurons form a regular grid in the (*θ,z*) space which represents the surface of the skin in cylindrical coordinates. At each timestamp, the decoded position of the presented stimulus is computed as the barycenter of the activities of the output neurons (see Methods).

### Contact detection and localization

**Fig. 3:**
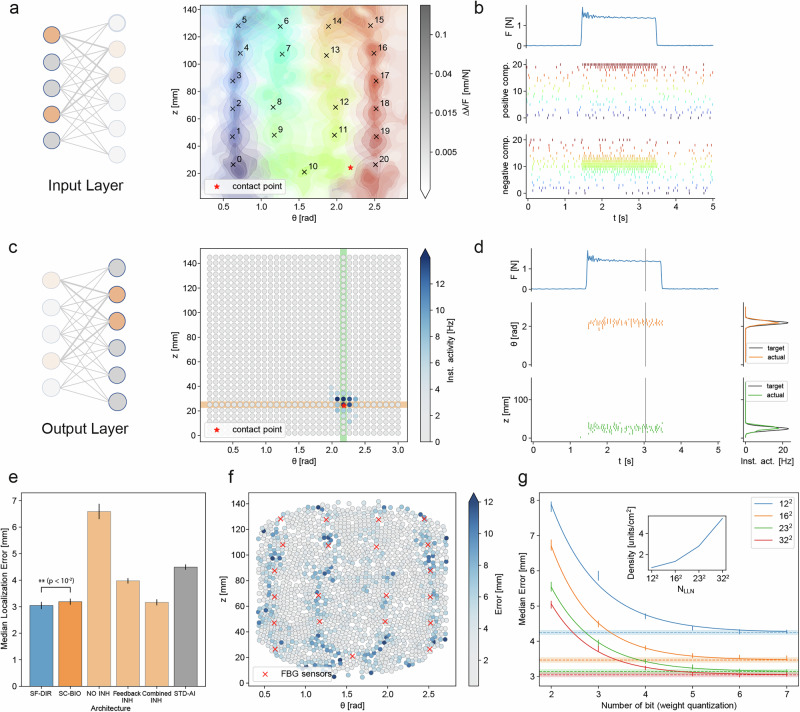
Localization and detection performance. **a** Contour plot of the intensity of the response of the 21 FBG transducers as a function of the position of the indentation showing that the sensitive regions of the transducers are significantly overlapped. **b** Temporal evolution of the force applied to the e-skin surface during indentation (top row) and spike trains of input layer neurons corresponding to the positive (middle row) and negative (bottom row) components of the FBG signals. **c** Instantaneous activity of the neurons in the output layer when an indentation is performed. **d** Temporal evolution of the applied force during an indentation (top row) and spike trains of the output layer neurons highlighted in subplot (**c**). The estimated position of the indentation is computed as the barycentre of the activities of the output neurons. **e** Medians of the localization error for the considered decoding strategies (blue: SF-DIR architecture; orange: SC-BIO architecture; light orange: three variants of the SC-BIO architecture: (i) without lateral inhibition (NO INH), (ii) with feedback inhibition replacing the original feedforward scheme (Feedback INH), and (iii) with both feedforward and feedback inhibition (Combined INH); gray: replication of the strategy described in ref. ^23^ and based on standard paradigms of AI). Error bars represent standard deviations of the median error computed via blocked bootstrap (one-tailed Wilcoxon signed-rank test on spatially blocked localization errors: *n* = 120, t = 4804, *p* = 0.001). **f** Scatter plot of the localization error over the surface of the skin. **g** Median localization error for different degrees of weight quantization and different numbers of output neurons. Error bars represent standard deviations of the median error computed via blocked bootstrap. Best fitted parameters and measure of goodness of fit for the exponential model are reported in Supplementary Tab. S1. Subpanels (a), (b), (c), (d), (f) and (g) report the results for the SF-DIR architecture.

When the surface of the artificial skin is stimulated, the wavelength shifts of the 21 FBG transducers are converted into spike trains by the input layer of the SNN (Fig. 3b) and then processed by the subsequent layers. After training, the instantaneous activities of the output neurons shape a Gaussian kernel around the stimulus position (Fig. 3c), enabling the estimation of the contact position (see Fig. 3d, Supplementary Movie S1 and Methods).

The median localization error performed by the presented tactile system is 3.05 ± 0.13 mm for the SF-DIR architecture and 3.19 ± 0.11 mm for the SC-BIO one (see Fig. 3e). Remarkably, both architectures significantly improve the performance of previous solutions^23^ based on standard approaches of AI, here reproduced and adapted to the presented artificial skin (see section 3.5 in Supplementary Information), which resulted in a median localization error of 4.49 ± 0.15 mm.

The error distribution for the SF-DIR network is uniform across the surface of the e-skin, with slightly larger errors observed in the regions above the path of the optical fiber (Fig. 3f). The characteristic dimension of spatial correlations of the localization error is ∼12 mm (see Supplementary Fig. S1). To account for this correlation, blocked techniques have been employed to estimate deviations in the median localization error via bootstrap and to statistically validate the performance comparison between architectures (see Methods). The median latency of the system is 26 ± 1 ms (see Supplementary Fig. S2), comparable with the characteristic delays of primary afferents in human somatosensory nervous system^3,5^.

To evaluate the feasibility of implementing our solution on devices with constrained memory and computational resources, we assessed how reducing the number of output layer neurons and quantizing synaptic weights affect localization performance. Our results show that the localization performance improves when more output neurons are employed, until the density of output neurons exceeds 4 neurons/cm^2^, when saturation occurs (see Fig. 3g). Further, the localization performances of the networks are robust to weight quantization, as the median localization error converges exponentially to the non-quantized level (Supplementary Tab. S1), achieving comparable results when using weights with a precision of 6 bits or higher (Fig. 3g).

Similar properties characterize the behavior of the SC-BIO architecture (see Supplementary Fig. S4) though its localization performance is lower than that of the SF-DIR network (one-tailed Wilcoxon signed-rank test on spatially blocked localization errors: *n* = 120, t = 4804, *p* = 0.001, effect size=0.28; see Fig. 3e and Methods).

To evaluate the functional relevance of the negative components of the FBG signals, which functionally mimic deformation-sensitive SA2 Ruffini mechanoreceptors (see Fig. 1c), we conducted a comparative study in which the SC-BIO and SF-DIR architectures were trained using only the positive component of the signal (see Fig. 1c). Under this condition, performance degraded substantially (median localization error: 5.5 ± 0.2 mm), confirming that inputs from broad, deformation-related receptive fields are crucial for accurate localization, and complementary to the more localized spatial information conveyed by the positive components.

Further, to investigate the functional relevance of lateral inhibition, we assessed the localization performance in the absence of inhibitory interneurons. This led to a marked degradation in localization accuracy, with a median localization error of 6.5 ± 0.2 mm (see Fig. 3e). We then explored alternative biologically plausible connectivity schemes including feedback inhibition. A first variant incorporating feedback-only inhibition performed worse than the SC-BIO model, with a median error of 3.98 ± 0.10 mm (see Fig. 3e). In contrast, the combination of feedforward and feedback inhibition resulted in a marginal improvement over the SC-BIO model, with a median localization error of 3.17 ± 0.09 mm (see Fig. 3e). These findings underscore the critical role of lateral inhibition in enhancing spatial acuity and suggest that the anatomical directionality of inhibition, feedforward rather than feedback, has functional implications. The inferior performance of the architecture lacking lateral inhibition confirms its necessity, while the limited benefit of combined inhibition suggests that feedforward pathways already provide near-optimal sharpening of the receptive field of output neurons.

Finally, to assess the generalization abilities of the system under multi-touch and more dynamic conditions, we evaluated its localization performance using a separate test dataset, with up to four simultaneous contact sites (see Supplementary Fig. S5b–e). Unlike previous experiments, indentations in this dataset were delivered manually, thereby introducing natural variability in force intensity and orientation (see Supplementary Fig. S5a). This led to a mean relative change rate during the stimulation (see methods) of 69%/s compared to the 29%/s characterizing the dataset collected with the robot and employed for training (see Supplementary Fig. S5f).

Under these conditions, the median localization errors for single-, double-, triple-, and quadruple-contact trials are 3.4 ± 0.4 mm, 6.1 ± 0.4 mm, 7.5 ± 0.9 mm, and 10.7 ± 0.9 mm, respectively (see Supplementary Fig. S5g).

Importantly, the employed decoding procedure (see Methods) does not require prior knowledge of the number of contact points, allowing the system to identify and localize multiple simultaneous stimuli based solely on the spiking activity of the output layer. Despite the increase in error in multi-pressure conditions, the network consistently operates in the super-resolution regime, achieving a 9.5× spatial resolution improvement over chance level. Further, localization in single-touch conditions matches the previously described performance on the more static, automatically collected dataset (see Fig. 3e and Supplementary Fig. S5g). These results demonstrate the ability of the architecture to generalize beyond training conditions, underscoring its robustness to more realistic, distributed and dynamic tactile stimuli.

### Energetic efficiency via bioinspired connectome

**Fig. 4:**
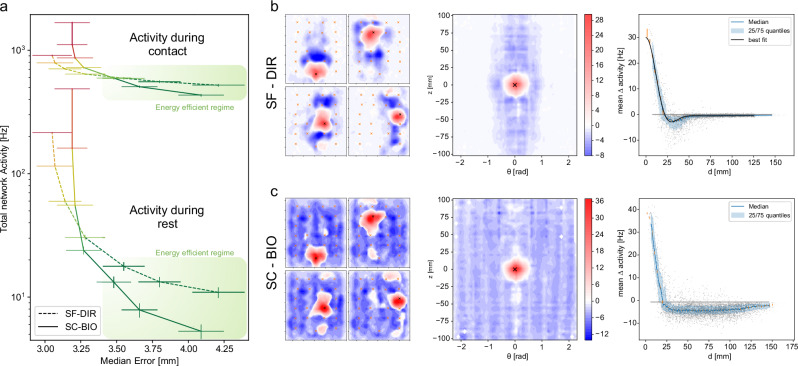
Biological insights on the early processing of the somatosensory system. **a** Total network spiking activity as a function of the median localization error for different degrees of energy efficiency constraint, in active and resting conditions. Lines represent median quantities and error bars represent their standard deviations estimated via blocked bootstrap. Note that in the energy efficient regime the bioinspired architecture has better localization performances. **b**, **c** Emergent functional connectivity properties between input and output neurons for the SF-DIR and SC-BIO architectures respectively: (left) connectivity patterns from four individual input neurons (black cross) and output neurons; (center) connectivity patterns averaged across input neurons mimicking SA1 mechanoreceptors; (right) projection over the radial dimension of the connectivity properties shown in the central panels: each dot represents a pair (input, output) neuron: the continuous blue-line and the shaded regions represent the median end interquartile values, respectively.

The adopted training protocol led to baseline currents of the input neurons that result in the emergence of spontaneous activity (i.e., activity in the absence of external stimuli, visible in Fig. 3b and in Supplementary Fig. S4b). As a consequence, both architectures in this configuration exhibit high spiking activities in both active (SC-BIO total activity = 1675 ± 25 Hz, SF-DIR total activity = 908 ± 12 Hz) and resting conditions (SC-BIO total activity = 487 ± 5 Hz, SF-DIR total activity 215 ± 3 Hz). Given the widespread and distributed nature of the tactile system, balancing performance and power consumption is crucial for both biological and technological systems^8,46^. To explore this trade-off, we repeated the training sessions with increasing degrees of regularization aiming at limiting the total spiking activity of the network (see Methods). We then analyzed the relationship between the performed localization errors of the two architectures and the spiking activities of the networks in active and resting conditions. As previously noted (see Fig. 3e), the SF-DIR architecture exhibits superior performance in the absence of constraints. Crucially, however, when the spiking activities of the two networks are limited (SC-BIO total activity in rest conditions = 5 ± 1 Hz, SF-DIR total activity during rest = 11 ± 1 Hz) and more energy-efficient regimes are considered, the performance curves of the two architectures intersect, with the bioinspired SC-BIO architecture eventually outperforming the SF-DIR architecture (see Fig. 4a and Supplementary Movie S2). These results are coherent with the notion that organisms in nature are not tailored to optimize absolute performance, but rather to allocate limited resources in a way that balances efficacy with efficiency in consideration of energy demands^46^.

### Emergent functional connectivity

A recent study^4^ showed that most projection neurons in the Dorsal Column Nuclei, particularly those targeting the somatosensory ventral posterolateral thalamus, exhibit small excitatory receptive fields with large region of surround inhibition. According to this study, these properties stem from the connectivity patterns between the primary afferents and the neurons in the Cuneate Nucleus, and they are pivotal for the correct transmission of fine spatial information.

To investigate the origins of these connectivity properties, we analyzed the post-training connectivity patterns characterizing the two considered architectures. To do so, we separately stimulated each neuron in the input layer and measured the response of the neurons in the output layer (see subpanels on the left of Fig. 4b, c). We then averaged the responses of the output neurons when different SA1-mimicking neurons of the input layer were selectively activated. Through this procedure, more generalized connectivity properties were revealed (see central and right subpanels of Fig. 4b, c). First, both networks exhibit a central excitatory bump, which reflects the sensitivity of SA1 primary afferents to local stimuli and establishes the core infrastructure of the somatotopic map between the skin and its representation in the Cuneate Nucleus.

On the other hand, the two architectures present markedly different strategies in the case of lateral inhibition. In the SF-DIR network the functional connectivity between mechanoreceptors and output neurons follows a distinct Mexican hat distribution (χ2 test: *n* = 16,  χ2= 21.38, p_val_ = 0.15) with organized inhibition primarily targeting intermediate-distance neurons (see Fig. 4b). In contrast, the bioinspired (SC-BIO) architecture features a more diffuse form of inhibition, affecting both intermediate and larger-distance neurons (Fig. 4c). While the Mexican hat topology is prevalent in many sensory modalities (e.g., in Lateral Geniculate Nucleus and V1 for vision^47^ and in primary afferents auditory neurons for hearing^48^), the functional connectivity of interneurons in the Cuneate Nucleus is poorly somatotopically arranged^39^ resulting, accordingly with experimental data in ref. ^4^, in larger regions of surround inhibition. Coherently with those observations, our results show that more spread forms of inhibition are distinctive of the bioinspired topology and that they spontaneously emerge through training. Similar patterns of lateral inhibition are also highlighted by the connectivity properties set up by SA2 mimicking neurons (see Supplementary Fig. S6).

### Hardware implementation on neuromorphic chip

**Fig. 5:**
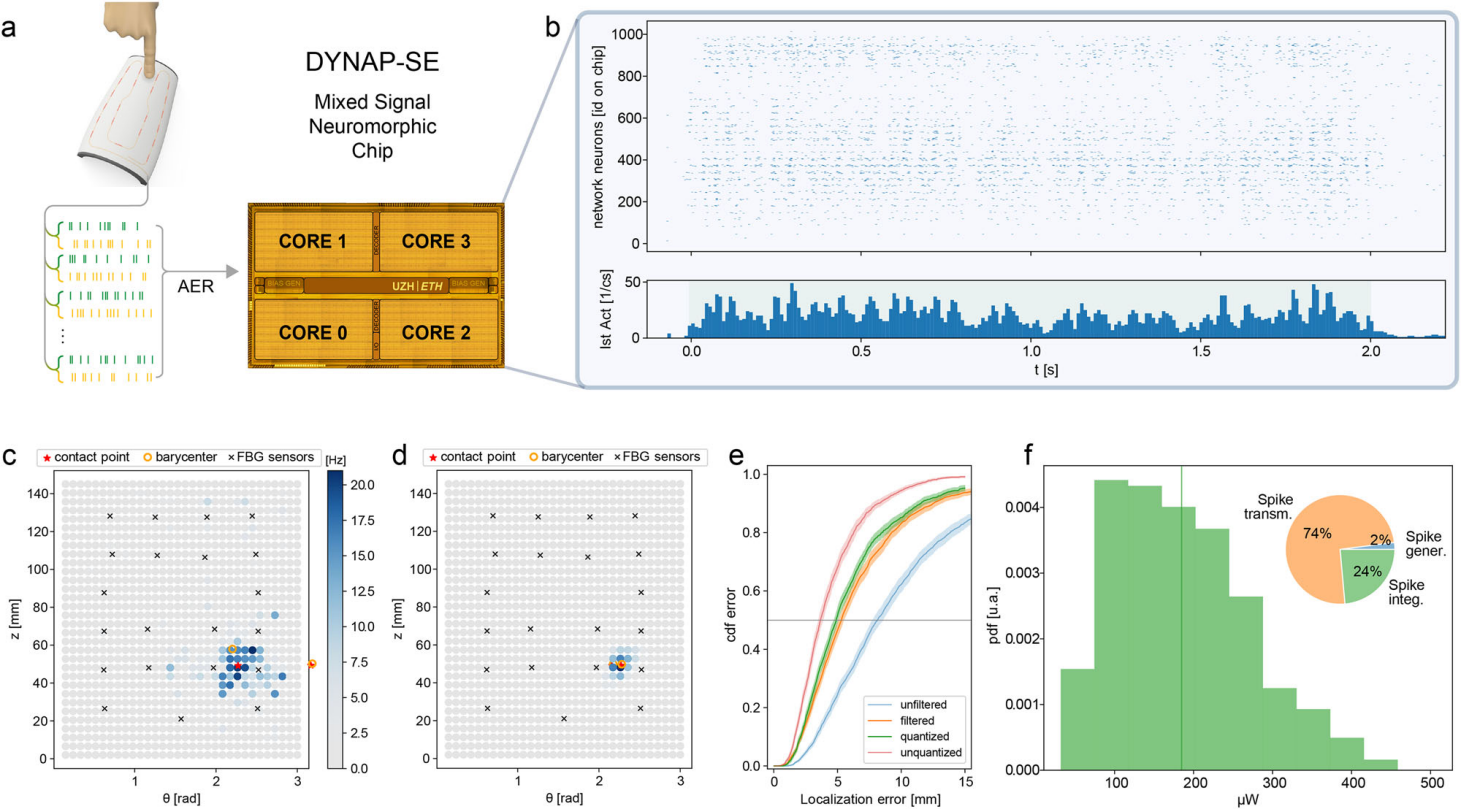
Implementation of the somatosensory spiking network on neuromorphic chip. **a** Design of the tactile system implemented on the DYNAP-SE processor: FBG signals are converted to spike trains and injected into the chip. **b** Raster plot of the activities of the neurons integrated on the chip. **c**, **d** Raw and filtered activities of the neurons in the output layer during indentation. **e** Cumulative distribution function of the performed localization error for the implementation on the neuromorphic chip with (orange) and without (blue) filter application, and for the software implementation with (green) and without (red) weight quantization. Shaded regions represent standard deviations of the CDF estimated via blocked bootstrap. **f** Histogram of the power consumption of the network during inference and relative contribution of spike generation, transmission and post-synaptic integration mechanisms to the total power consumption of the chip.

To evaluate the robustness and feasibility of implementing the proposed tactile system on low-power neuromorphic hardware, we adapted the SC-BIO network to be integrated on the DYNAP-SE processor^39^ (see Fig. 5a). The DYNAP-SE chip is a mixed-signal neuromorphic processor comprising 4096 adaptive exponential analog neurons divided into 16 cores. Each neuron presents four synapses, two inhibitory and two excitatory, with exponential decaying currents. Each of these synapses can be individually selected for each presynaptic neuron, though their weights are shared among neurons. Therefore, the 2-bit quantized version of our network can be implemented on the chip (see Methods). To mitigate the effect of spurious spikes in the output layer (see Fig. 5c), a local online spatial filtering is applied at the output layer, before computing the barycenter of neuron activity (see Fig. 5d and Methods). The resulting median localization error of the bioinspired network in the energy-efficient regime is 5.33 ± 0.15 mm, resulting in only a 8% performance degradation compared to the software results in the same condition (Fig. 5e and Supplementary Movie S3).

In the proposed implementation, most of the neural computation (95% of neurons—see Methods) is integrated on the DYNAP-SE processor, obtaining a 185 μW median power consumption of the neuromorphic chip during inference (see Fig. 5f and Methods)^49^. Notably, the largest portion of this power (74%) is employed for mechanisms associated with spike routing and broadcasting across the processor. By implementing neuronal dynamics directly in analog circuits, the chip eliminates the need for explicit numerical integration of differential equations. Furthermore, the hardwired integration of synaptic circuits within the processing units, minimizes instruction handling and data transfer overhead. These architectural choices account for the high efficiency of the chip during inference, and showcase even greater advantages when no stimuli are applied. Indeed, during resting conditions, the sparse activation of neurons, combined with the event-driven and in-memory-computing architecture, reduces power consumption to just 8.7 µW (see Supplementary Fig. S7). In contrast, a more conventional digital implementation of the same network, which simulates rather than emulates neural dynamics, would require approximately 10 million floating-point operations per second to continuously integrate the differential equations. Running this computational load on modern ultra-low power ARM processors would require a clock frequency of approximately 50 MHz and would therefore result in a power consumption in the order of mW, in both active and resting conditions.

## Discussion

Following the recent advancements in robotics and the multiple capabilities provided by somatosensory perception in biological systems, numerous studies investigated materials and transducers to engineer sensorized e-skins^22,50^, reconstruct their deformation map^26^ and achieve high levels of super-resolution^25^ through deep artificial neural networks. Meanwhile, complementary studies integrated the design of tactile sensors with AI to address object and texture recognition tasks^24,27,51^. In particular, significant results were achieved by means of SNNs, which enabled the decoding of touch features with high performance and very low latencies^30^.

In this work, we combined the advancements in the design of e-skins with the efficient spatio-temporal processing capabilities enabled by SNNs to engineer a bioinspired tactile architecture aimed at the detection and localization of external stimuli. The resulting solution combines flexibility^18,19^, modularity^51^, balanced sensor density^25,26^ and minimal wiring^23^, which are key properties for scaling sensing systems to full robot coverage. In addition, at the core of the proposed system is a SNN, which outperforms previously published results^23^ based on standard paradigms of AI improving localization accuracy by 32%. The proposed framework also generalizes effectively to more complex tactile inputs. When tested on manually delivered stimuli involving multiple simultaneous contact points, the system maintained high localization accuracy and a strong detection rate. Importantly, these stimuli were not included in the training phase, demonstrating robustness and adaptability to real-world tactile scenarios involving distributed and dynamic tactile interactions. As a leap forward over state-of-the-art solutions, our tactile system can be successfully implemented on asynchronous neuromorphic processors with analog neurons. This resulted from an optimized association procedure between computationally- and hardware- emulated neurons, which accounts for the actual dynamical properties of the physical substrate. As a consequence, the presented hardware demonstrator more closely emulates biological circuits, which are heavily affected by large degrees of natural heterogeneities, while concurrently supporting ultra-low power and parallel processing, rapid responsiveness and distributed sensitivity.

While our system currently relies on a benchtop optical interrogator for the readout of FBG tactile transducers, recent advancements in integrated photonics are enabling the development of miniaturized, low-power optical readout systems^52^ anticipating the feasibility of compact solutions suitable for scalable e-skin applications.

Overall, these findings support the broader perspective that highly effective sensory processing is possible in analog substrates, even in the absence of deterministic timing precision, and foster the development of hybrid architectures harnessing the efficiency of analog computing^41,42^.

The design of the proposed system balances bio-emulative and engineering solutions. Interestingly, the comparison between the two considered neuronal architectures revealed that mimicking the structure of the somatosensory system was critical in the identification of an optimal trade-off between performance and power consumption. While some might argue that the alternative SF-DIR architecture cannot be observed in nature - since neurons typically either excite or inhibit postsynaptic neurons - this limitation can be addressed by duplicating each presynaptic neuron, with one dedicated to excitation and the other to inhibition. This approach is applied in other sensor modalities, as for example in vision (with bipolar ON–OFF cells responding to complementary changes in light^53^), and could therefore also be employed in the tactile system. Nonetheless, organisms in nature have limited resources, which they must allocate optimally among vital functions like growth, reproduction, and survival to enhance their overall fitness^46^. Due to its direct impact on these functions, energy efficiency represents a critical aspect^46^. Based on the developed model of the early somatosensory system, our analysis suggests that the SC-BIO architecture might have been favored for somatosensory perception because of its superior performance in energy constrained conditions. Indeed, in the considered context of touch perception, the somatosensory system likely sacrificed a limited fraction (15% in this study) of tactile localization in exchange for a significant reduction (more than an order of magnitude, in resting conditions) of the overall spiking activity of the network. Complementary to these results, the proposed comparison between different inhibitory topologies—including feedforward, feedback, and hybrid connectivity motifs— quantitatively evaluates their functional roles in tactile sensory processing. Rather than just replicating biological structure for plausibility^38^, this investigation tests alternative circuit hypotheses within a principled framework that integrates the formulation of physiological hypotheses, the design of simplified computational models, and experimental validation in a realistic tactile scenario. By leveraging this convergence, our work provides mechanistic insights into early somatosensory processing and offers a quantitative computational perspective on why specific neural architectures might have been selected, or disfavored, in the early processing of somatosensory system. Specifically, our work underscores the critical role of lateral inhibition in enhancing spatial acuity and suggests that the anatomical directionality of inhibition, feedforward rather than feedback, has functional implications. These results align with general principles of sensory system organization, where feedforward connectivity is typically functionally dominant in the early stages of sensory processing across multiple modalities, including audition^54^, touch^3,7^ and vision^55,56^ systems. These pathways prioritize rapid and reliable transmission of stimulus features, enabling primary afferents to convey spatio-temporally precise input to higher-order areas with minimal latency and high fidelity.

Balancing bio-emulative and engineering solutions, the presented tactile system thus effectively decodes applied stimuli, presents low-power consumption and fosters distributed computing through mixed signal architectures. These combined features make the proposed system ideal for next-generation robots, which safely cooperate with humans relying on fast, reliable, distributed and energy efficient tactile sensing.

## Methods

### FBG-based e-skin and dataset collection

The e-skin is designed to replicate the shape of a human forearm and consists of a soft silicone substrate that integrates a single optical fiber with 21 FBG sensing elements^37,57^ (Fig. 1a). These sensors are inscribed within the fiber core as local periodic variations in its refractive index. When illuminated by broadband light, each FBG reflects a narrow portion of the received spectrum, which is centered at a characteristic wavelength, known as the Bragg wavelength (λ_B_), and defined as:

1 $${\lambda }_{B}=2{n}_{{eff}}\varLambda \,$$

where *n*_*eff*_ is the local effective refractive index of the fiber and *Λ* is the grating pitch^37^.

Variations in these physical properties due to external stimuli cause a shift in the reflected peak^37^ (Fig. 1b), enabling mechanical sensitivity. This sensitivity, along with other advantages, such as a small form factor, immunity to electromagnetic interference, and the ability to integrate multiple sensors in a single fiber, makes FBG-based transducers well-suited for touch sensing devices.

In the proposed e-skin, the optical fiber is embedded in the silicone substrate through a three-step fabrication process. First, a mold is realized to pour the silicone (Dragon Skin 10, Smooth-On Inc.) in and let it polymerize as a solid soft layer with the desired shape. Then, the optical fiber is inserted and glued inside a predefined groove on the patch. Finally, the patch is covered with an additional layer of silicone using a second custom mold. Embedding the fiber in this rubber-like substrate enhances the robustness of the e-skin and facilitates the propagation of the applied stimuli, enhancing the cross-talk between the FBG sensors.

To collect the dataset, the e-skin surface was modeled as a triangular mesh, whose triangle centroids were extracted as target indentation points. For each triangle, the normal direction was computed and used to define the orientation of the end-effector of an indenting robot. In more details, a bimanual robotic platform, consisting of two Racer 5 L anthropomorphic arms (Comau, Italy), was used to collect the dataset. The two arms were equipped with a plate to hold and orient the e-skin and an elastic indenting probe to apply the stimuli, respectively. The indentations were performed in random order at the predefined target locations and consisted in quasi-static stimuli applied through a force-feedback control loop. A 6-axis load cell (Nano 43, ATI Industrial Automation, USA) was mounted between the end-effector of the robot and the indenting probe to measure force and torque values. The indentation protocol was automated using a dedicated LabVIEW routine (National Instruments, USA), developed to control the two robotic arms and store data.

The resulting dataset consisted of 2026 matrices, each including: (i) wavelength variations from the 21 FBG sensors, measured through a commercial optical interrogator (FBG-Scan 904, FBGS International NV, Belgium); (ii) the Cartesian coordinates of the indentation points; and (iii) force and torque data from the 6-axis load cell. The FBG sensor data were then corrected for offsets to obtain the wavelength shift (Δλ), and the magnitude of the applied load was computed for each indentation.

To evaluate the properties of the receptive fields of the positive and negative components of the Δλ signals, we analyzed the responses of the sensors as a function of the relative position between them and the performed stimuli (see Fig. 1c, d). The characteristic dimensions of the receptive field of the two components have been assessed by modeling their mean intensity, ${\mathbb{E}}$[R^(+,-)^], as an exponential function of the distance *d* between each sensor and the performed stimuli:

2 $${\mathbb{E}}\left[{R}^{\left(+, -\right)}\left(d\right)\right]={R}_{0}^{\left(+,-\right)}\exp \left(-\frac{d}{{d}_{0}^{\left(+,-\right)}}\right)$$

And then fitting the values of R_0_^(+,-)^ and d_0_^(+,-)^ separately for the two components.

To assess the degree of regularity of the response we employed the mean value of the Coefficient of Variation:

3 $${{\mathbb{E}}}_{d}\left[C{V}^{\left(+,-\right)}\left(d\right)\right]={{\mathbb{E}}}_{d}\left[\frac{{{\rm{std\; dev}}}\left({R}^{\left(+,-\right)}\left(d\right)\right)}{{\mathbb{E}}\left[{R}^{\left(+,-\right)}\left(d\right)\right]}\right]\, \quad d\in \left[0,\,3{d}_{0}^{(+,-)}\right]$$

The value of CV^(+,-)^(d) is representative of the relative variability of the FBG sensor responses when considering stimuli performed at a given distance $d$. Consequently, lower values of CV(d) indicate higher degrees of cylindrical symmetry of the sensor responses.

To evaluate the system in multi-touch conditions, an additional dataset was collected using manual indentations applied to the surface of the e-skin. A custom-designed mask with a 9 × 9 grid of evenly distributed tactile stimulation sites was used to define possible contact locations. For each trial, one to four simultaneous contact sites were randomly selected. A total of 300 unique configurations (75 per condition) were applied in random order to ensure a diverse and representative set of multi-touch configurations. Each indentation lasted approximately 3 s and was applied by hand, introducing natural variability in force intensity and orientation.

To quantitatively assess the dynamical properties of this new dataset compared to previous experiments, we analyzed the rate of relative change of the input signals under both conditions. Specifically, for each indentation $i$, the rate of relative change (rrc) was computed as:

$${{\rm{rrc}}}\left(i\right)=\,\frac{1}{{N}_{{FBG}}}{\sum }_{j=1}^{{N}_{{FBG}}}{{\mathbb{E}}}_{t}\left[\frac{\left|\frac{d{s}_{{ji}}}{{dt}}\left(t\right)\right|}{\left|{s}_{{ji}}\left(t\right)\right|}\right]\,$$

where ${s}_{{ji}}(t)$ is the signal of the *j*-th FBG signal during the *i*-th indentation and ${{\mathbb{E}}}_{t}[\cdot ]$ denotes the temporal average over the duration of the indentation.

### Neuronal dynamics and inputs to the SNN

All neurons in the SNN are modeled as current based Leaky Integrate and Fire neurons with exponentially decaying currents:

$${C}_{m}\frac{{{\rm{d}}}V}{{{\rm{d}}}t}=-{g}_{L}\left(V-{E}_{L}\right)+{I}_{{ex}}+{I}_{{in}}+{I}_{0}+{I}_{{ext}}\,$$

4 $${\tau }_{{ex}}\frac{{{\rm{d}}}{I}_{{ex}}}{{{\rm{d}}}t}=-{I}_{{ex}}+{\tau }_{{ex}}{\sum }_{{{\rm{ex\; input}}}\,i}{\omega }_{i}\delta \left(t-{t}_{i}-{t}_{r}\right){\tau }_{{in}}\,$$

$${\tau }_{{in}}\frac{{{\rm{d}}}{I}_{{in}}}{{{\rm{d}}}t}=-{I}_{{in}}+{\tau }_{{in}}{\sum }_{{{\rm{in\; input}}}\,i}{\omega }_{i}\delta \left(t-{t}_{i}-{t}_{r}\right)\,$$

where *V* is the membrane potential, *I*_*ex*_ and *I*_*in*_ are the excitatory and inhibitory synaptic currents, *I*_*0*_ and *I*_*ext*_ are the neuron-specific baseline and external currents respectively. Whenever the membrane potential *V* reaches the threshold *V*_*th*_ an output spike is emitted, and the potential is reset to *V*_*res*_. Following the emission of a spike, the membrane potential *V* of the neuron is forced to the reset-value for a refractory period with duration *t*_*ref*_. The values of the parameters have been fixed to:

$${C}_{m}=40{{\rm{pF}}}\,\quad{g}_{L}=2{{\rm{nS}}}\,\quad{\tau }_{{ex}}=8{{\rm{ms}}}\,\quad{\tau }_{{in}}=4{{\rm{ms}}}$$

$${E}_{L}={V}_{{res}}=-70{{\rm{mV}}}\,\quad{V}_{{th}}=-50{{\rm{mV}}}\,\quad{t}_{r}=2{{\rm{ms}}}\,\quad{t}_{{ref}}=2{{\rm{ms}}}$$

The neuronal dynamics has been integrated using the Euler method with a fixed step size of 1 ms. In addition to the spike inputs from presynaptic neurons, each neuron receives supplementary excitatory stimuli modeled as independent Poisson processes with a constant mean *ν*_*ext*_ (i.e., the number of input spike in a time interval with duration $T$ is distributed according to a Poisson distribution with mean *ν*_*ext*_ *T*). Particularly we set the values:

$${\omega }_{{ext}}=2{\rm{pA}},\quad{\nu }_{{ext}}=1{\rm{kHz}}$$

Consequently, the excitatory input current to each neuron presents an additive noisy component ξ(t) with the following properties:

5 $${\mathbb{E}}\left[\xi \left(t\right)\right]={\omega }_{{ext}}{{\nu }_{{ext}}\tau }_{{ex}}=16{\rm{pA}}\; \quad {\rm{std \ dev}}\left[\xi \left(t\right)\right]={\omega }_{{ext}}\sqrt{{{\nu }_{{ext}}\tau }_{{ex}}}=4{\rm{pA}}\,$$

In addition to this current, neurons in the input layer receive the time-varying signals Δλ_i_(t) generated by the 21 FBG transducers. In particular, for each time *t* and for each FBG sensor *i*, the positive and negative components of the Δλ_i_(t) signals are extracted:

6 $$\Delta {\lambda }_{i}\left(t\right)\,\mapsto \,\left\{\begin{array}{c}{\Delta {{\rm{\lambda}}} }_{i}^{\left(+\right)}\left(t\right)={\rm{ReLU}}\left(\Delta {{\rm{\lambda}}} \left(t\right)\right)\\ {\Delta {{\rm{\lambda}}} }_{i}^{\left(-\right)}\left(t\right)={\rm{ReLU}}\left(-\Delta {{\rm{\lambda}}} \left(t\right)\right)\end{array}\,\right.$$

and transformed through a logarithmic transformation ${f}^{\left(\cdot \right)}$:

7 $$\left\{\begin{array}{c}{\Delta {{\rm{\lambda}}} }_{i}^{\left(+\right)}\left(t\right)\\ {\Delta {{\rm{\lambda}}} }_{i}^{\left(-\right)}\left(t\right)\end{array}\right.\mapsto \left\{\begin{array}{c}{I}_{i}^{\left(+\right)}\left(t\right)={f}^{\left(+\right)}\left({\Delta {{\rm{\lambda}}} }_{i}^{\left(+\right)}\left(t\right){\rm{;LF}}\right)\\ {I}_{i}^{\left(-\right)}\left(t\right)={f}^{\left(-\right)}\left({\Delta {{\rm{\lambda}}} }_{i}^{\left(-\right)}\left(t\right){\rm{;LF}}\right)\end{array}\,\right.$$

where ${{\rm{LF}}}$ is a parameter regulating the degree of non-linearity of the transformation (see section 3.2 in the Supplementary Information). As the positive and negative components of Δλ_i_ present different sensitivity properties (see Fig. 1), the resulting current I_i_^(+)^ and I_i_^(-)^ are fed as external currents *I*_*ext*_ into separate neurons of the input layer.

### Derivation of the stimuli coordinates (single touch)

The estimated coordinates *θ(t)* and *z(t)* are computed starting from the activities ${ \{{y}_{i}^{(L)}\} }_{i=1}^{{N}^{(L)}}$ of the output layer. In particular:

- Given *τ*_*out*_ = 100 ms, *δt* = 1 ms, the quantity:
  8 $${L}_{i}\left({t}_{n}=n\delta t\right)=\,\frac{1}{{\tau }_{{out}}}\left({e}^{-\frac{x}{{\tau }_{{out}}}} * {\sum }_{{t}_{i}}\delta \left(x-{t}_{i}\right)\right)\left(t\right)\,$$
  is computed for each output neuron;
- For all *t*_*n*_, the neurons with higher ${L}_{i}\left({t}_{n}\right)$ are considered to compute the *instantaneous* barycenter $\left(\widetilde{\theta }\left({t}_{n}\right),\,\widetilde{z}\left({t}_{n}\right)\right):$
  9 $$\widetilde{\theta }\left({t}_{n}\right)=\,\frac{{\sum }_{j\in J\left({t}_{n}\right)}{L}_{j}\left({t}_{n}\right){\,\theta }_{j}}{{\sum }_{j\in J\left({t}_{n}\right)}{L}_{j}\left({t}_{n}\right)}\,\widetilde{z}\left({t}_{n}\right)=\,\frac{{\sum }_{j\in J\left({t}_{n}\right)}{L}_{j}\left({t}_{n}\right){\,z}_{j}}{{\sum }_{j\in J\left({t}_{n}\right)}{L}_{j}\left({t}_{n}\right)}\,$$
  where $J\left(t\right)=\left\{j\in \left[1,{N}^{\left(L\right)}\right]{{\rm{s}}}.{{\rm{t}}}.\,{L}_{j}\left(t\right) > {{\rm{quantile}}}\left(\left\{{L}_{k}\left(t\right) > 0\right\},0.9\right)\right\}$;the estimated coordinates$\,\left(\theta \left({t}_{n}\right),{z}\left({t}_{n}\right)\right)$ are computed considering the *unbiased* exponential moving average of $\left(\widetilde{\theta }\left({t}_{n}\right),\,\widetilde{z}\left({t}_{n}\right)\right)$:

10 $$\theta :\left\{\begin{array}{c}\hat{\theta }\left({t}_{n}=n\delta t\right)={\alpha }_{{out}}\hat{\theta }\left({t}_{n-1}\right)+\left(1-{\alpha }_{{out}}\right)\,\widetilde{\theta }\left({t}_{n}\right)\,\\ \theta \left({t}_{n}=n\delta t\right)=\frac{\hat{\theta }\left({t}_{n}\right)}{1-{\alpha }_{{out}}^{n+1}}\end{array}\right.$$

11 $$z:\left\{\begin{array}{c}\hat{z}\left({t}_{n}=n\delta t\right)={\alpha }_{{out}}\hat{z}\left({t}_{n-1}\right)+\left(1-{\alpha }_{{out}}\right)\,\widetilde{z}\left({t}_{n}\right)\,\\ z\left({t}_{n}=n\delta t\right)=\frac{\hat{z}\left({t}_{n}\right)}{1-{\alpha }_{{out}}^{n+1}}\end{array}\,\right.$$

where ${\alpha }_{{out}}=0.9975$ and the values $\hat{\theta }\left({t}_{n=-1}\right)$ and $\hat{z}\left({t}_{n=-1}\right)$ are initialized to zero.

The detection and localization of the stimuli is triggered whenever the mean activity of the output layer steadily exceeds 0.02 Hz (see Supplementary Movies S1, S2 and S3).

### Derivation of the stimuli coordinates (multi-touch)

As in the single-touch scenario, detection and localization under multi-touch conditions is driven by the spiking activity of the neurons in the output layer. Specifically, the following iterative procedure is applied: the neuron with the highest activity is identified; if its activity exceeds a predefined threshold of 3 Hz, its corresponding position is registered as an active contact point. To avoid redundant detections, the activity of this neuron and its neighbors is suppressed using a Gaussian kernel with fixed width $\sigma=1.5$ cm. The procedure is repeated iteratively until no neuron exceeds the threshold. Importantly, this procedure enables localization under multi-touch conditions without prior knowledge of the number of active contact points. Missed contacts have been penalized in the computation of the localization error by assigning the center of the skin as default estimated position.

### Training algorithm

The learning procedure employed in this study is based on stochastic gradient descent. Although it requires error backpropagation from the output layer, it reduces the computational costs associated with standard BPTT-based algorithms^58–63^ by avoiding the storage of the hidden states and the exact spike times of the neurons during the forward pass. Given a time interval of duration $W$, we define the instantaneous activity *y*_*j*_^*(l)*^*(t)* of neuron $j$ in layer $l$ as the number of spikes emitted by the neuron within the time interval $\left[t-\frac{W}{2}{;t}+\frac{W}{2}\right]$. In both networks, each neuron *i* in the output layer is associated with a specific position (θ_i_,z_i_) in the (θ,z) space, representing the skin surface in cylindrical coordinates. Each of these neurons is trained to reproduce a target activity *y*_i_^*^(t) that shapes a Gaussian kernel around the actual stimulus position (θ_0_(t),z_0_(t)):

12 $${y}_{i}^{*}\left(t\right)\propto \exp \left(-\frac{{d\left({\theta }_{i} \cdot {z}_{i};\,{\theta }_{0}\left(t\right),\,{z}_{0}\left(t\right)\right)}^{2}}{2{\sigma }^{2}}\right)$$

where $d\left({\theta }_{i}.{z}_{i}{;}{\theta }_{0},\,{z}_{0}\right)$ is the distance between (*θ*_*i*_*,z*_*i*_) and (*θ*_*0*_*,z*_*0*_) over the surface of the skin and *σ* is an hyperparameter regulating the size of the Gaussian kernel. The distance between two points on the surface of the e-skin is estimated as their distance over the surface of the best fitting cone.

To train the networks, the weights ${\omega }_{{ji}}^{\left(l-1\right)}$ from neuron $i$ in layer $l-1$ to neuron $j$ in layer $l$ and the baseline currents ${{I}_{0,j}^{(l)}=I}_{j}^{\left(l\right)}$ are updated every 10 indentations by integrating the instantaneous corrections ${{\rm{d}}}{\omega }_{{ji}}^{\left(l-1\right)}\left(t\right)$ and ${{\rm{d}}}{I}_{j}^{\left(l\right)}\left(t\right)$:

13 $$\Delta {\omega }_{{ji}}^{\left(l-1\right)}={\sum }_{n}d{\omega }_{{ji}}^{\left(l-1\right)}\left(n{t}_{s}\right)\,\,d{\omega }_{{ji}}^{\left(l-1\right)}\left(n{t}_{s}\right)=-\lambda {\sum }_{n} m\left({I}_{j}^{\left(l\right)}\left(n{t}_{s}\right)\right)H\left({y}_{j}^{\left(l\right)}\left(n{t}_{s}\right)\right){\tau }_{{ji}}^{\left(l\right)}{y}_{i}^{\left(l-1\right)}\left(n{t}_{s}\right){\widetilde{E}}_{j}^{\left(l\right)}\left(n{t}_{s}\right)\,$$

14 $$\Delta {I}_{j}^{l}={\sum }_{n}{{dI}}_{j}^{\left(l\right)}\left(n{t}_{s}\right)\,\,d{I}_{j}^{\left(l\right)}\left(n{t}_{s}\right)=-\lambda {\sum }_{n} m\left({I}_{j}^{\left(l\right)}\left(n{t}_{s}\right)\right)H\left({y}_{j}^{\left(l\right)}\left(n{t}_{s}\right)\right)W{\widetilde{E}}_{j}^{\left(l\right)}\left(n{t}_{s}\right)$$

where:

- $\lambda$ is the learning rate;
- the values of ${t}_{s}$ and $W$ are fixed to 100 and 500 ms respectively;
- ${\tau }_{{ji}}^{\left(l\right)}$ is the characteristic decay time of ${I}_{j}^{\left(l\right)}\left(t\right)$: hence ${\tau }_{{ji}}^{\left(l\right)}={\tau }_{{ex}}$ if ${\omega }_{{ji}}^{\left(l-1\right)} > 0$ else ${\tau }_{{ji}}^{\left(l\right)}={\tau }_{{in}}$;
- $m\left(x\right)$ is the slope factor of the FI curve of the neurons;
- $H\left(x\right)$ is the Heaviside step function ($H\left(x\right)=1$ if $x > 1$ and $H\left(x\right)=0$ otherwise);
- ${\widetilde{E}}_{j}^{\left(l\right)}\left(t\right)$ is the generalized instantaneous error of neuron ${j}$ in layer $l$ (see section 3.3 in Supplementary Information).

Further, the following regularization techniques are applied:

- the learning rate is decreased linearly as a function of the error variation in consecutive epochs $\delta {E}_{{{\rm{epoch}}}}$:
  15 $$\,\lambda\; \mapsto\, \lambda \left(\delta {E}_{{\rm{epoch}}};{\lambda }_{m},{\lambda }_{M},\delta {E}_{m},\delta {E}_{M}\right)=\,\left\{\begin{array}{c}{\lambda }_{m}\quad {\rm{if}} \ -\delta {E}_{{epoch}} < \delta {E}_{m}\hfill\\ {\lambda }_{M}\quad {\rm{if}} \ -\delta {E}_{{epoch}} > \delta {E}_{M}\hfill\\ \,\frac{{\lambda }_{M}-{\lambda }_{m}}{\delta {E}_{M}-\delta {E}_{m}}\left(-\delta {E}_{{\rm{epoch}}}-\delta {E}_{m}\right)+{\lambda }_{m}\quad{\rm{otherwise}}\end{array}\,\right.$$
- L2 regularization is applied: the updates of the weights given by Eq. (13) are corrected by a weight-decay term:
  16 $$\Delta {\omega }_{{ij}}\,\mapsto {\,\Delta \omega }_{{ij}}-{\kappa }_{{decay}}{\omega }_{{ij}}$$
- The Heaviside step function is modified with a *leaky* component:
  17 $$H\left(x\right)=\left\{\begin{array}{c}\,1\quad\quad \ {\rm{if}} \ x > 0\,\hfill\\ {h}_{{leak}} \quad {\rm{otherwise}}\,\end{array}\,\right.$$

The adopted learning protocol can be derived from the principle of error-minimization and applying stochastic gradient descent (see section 3.3 in Supplementary Information).

To limit the overall network activity and investigate the performance of the network in different regimes of power efficiency, the following strategies have been implemented:

- an upper bound is introduced for the value of the external baseline current to all neurons:
  18 $$\forall \,j,\,l\,\quad{I}_{j}^{\left(l\right)} < \,{I}_{{MAX}}^{\left(l\right)}\,$$
- the value of the L2 regularization parameter ${\kappa }_{{decay}}$ has been modulated by the mean activity ${y}^{\left(l\right)}$ of the postsynaptic population:
  19 $${\kappa }_{{decay}}=\,{\kappa }_{{decay},0}\left(1+\frac{{M}_{{decay}}}{1+{e}^{-{y}^{\left(l\right)}/{s}_{{decay}}}}\right)$$
- the number of interneurons in the SC-BIO network has been linearly decreased.

A summary of the values employed for all the described parameters is provided in the supplementary information (see Supplementary Tab. S5).

### Statistical analyses

The performance of the different networks has been evaluated employing 5-fold validation and comparing the distributions of the median localization error performed in the test sets. The same 5-fold testing have been employed for all the considered networks and for every degree of energy efficiency, ensuring equal conditions. P-values are computed applying the non-parametric Wilcoxon Signed-Rank Test and comparing the localization error performed by the different networks when the same stimuli are presented. To account for spatial correlations, we grouped the indentations into blocks based on their positions. The block size has been chosen as the smallest size reaching a plateau in the computation of the standard deviation of the median error estimated via blocked bootstrap (see Supplementary Fig. S1). Consistently, error measurements for median quantities have been estimated using blocked bootstrap with the same block size. Effect sizes have been estimated using the *r* = z/sqrt(n) statistic, where *z* is the standardized test statistic and *n* is the total number of observations. To evaluate the goodness of fit for localization performance under different degrees of weight quantization, p-values have been computed using the chi2 statistics on the residuals between the observed localization errors and an exponentially decreasing model:

$${{\rm{loc}}}.{{\rm{error}}}\left({n}_{{bit}}\right)=a{\mathrm{exp}}\left(-\frac{{n}_{{bit}}}{{\tau }_{{bit}}}\right)+c$$

where $c$ is the localization error in the case of continuous weights and $a$ and ${\tau }_{{bit}}$ are free parameters. Due to the presence of autocorrelation in the localization errors across consecutive values of ${n}_{{bit}}$ (see Supplementary Fig. S3), the effective number of independent samples ${N}_{{eff}}$ has been adjusted according to ${N}_{{eff}}={N}_{{samples}}(1-{e}^{-1/{\tau }_{{ACF}}})/(1+{e}^{-1/{\tau }_{{ACF}}})$ when performing the chi2 test.

### Software implementation of the SNN

The code for the simulation and training of the SNN has been developed in C + + and is structured in such a way that general networks of supported point neurons can be implemented and trained with the adopted training algorithm. In combination with this code, a Python3 module has been implemented in order to handle the training process and import and preliminarily analyze the results of the simulations (see Code Availability statement for details).

### Implementation on the DYNAP-SE chip

The employment of SNN^29,64,65^ enables high degrees of power efficiency when implemented on neuromorphic processors^29^. Because of this reason, various neuromorphic chips have been developed^66–70^, with distinct approaches in terms of modeling and integration of neuronal dynamics, synchronous versus asynchronous computation, digital/analog implementations, degrees of brain computing emulation and memory requirements and management^71^. Among these possibilities, asynchronous analog solutions exploit the diffusion mechanism of electrons in CMOS transistors operating in the subthreshold regime to emulate ion-flows in neuronal ion-channels^72,73^. This approach enables the creation of compact and low-power neuromorphic circuits that mimic the biophysics of neurons and synapses^74,75^. While bolstering highly power efficient computation, analog design is inherently sensitive to noise and neuron-to-neuron mismatch^40^. These properties can potentially lead to severe performance degradation when transferring from software to hardware implementations. To test the robustness of the proposed algorithm to these properties, we adapted the SC-BIO network in the energy efficient regime to be implemented on the DYNAP-SE neuromorphic processor^39^.

The DYNAP-SE chip is a low power multi-core processor with mixed-signal, asynchronous circuits, fabricated using a standard 0.18 μm 1P6M CMOS technology. The chip implements 4096 adaptive exponential LIF neurons^76^, each provided with 4 binary synapses. Both neuron and synaptic dynamics are integrated through dedicated analog circuits employing CMOS transistor in the subthreshold regime. The efficacies of the four synapses and the input baseline current of the neurons are tunable but shared among neurons.

To account for the hardware neuronal dynamics —which includes a non-tunable, voltage-dependent exponential drift— we fine-tuned the 2-bit quantized weight values and baseline input current through training with chip in the loop (see section 3.6 in Supplementary Information).

Further, to leverage neuron heterogeneities, we optimized the process of software-to-hardware neuron association, by matching each simulated output neuron to the one in the processor which best mimics its behavior (see section 3.6 in Supplementary Information).

Finally, to mitigate the effect of spurious spikes in the output layer, we applied a local online spatial filtering to the activities of the output layer, before computing their barycenter:

20 $$\forall i:\,{y}_{i}\to {y}_{i}\cdot \,\frac{{\sum }_{j \; {{\rm{nearest}}}\, {{\rm{neig}}}\;. {{\rm{of}}} \; i}\,{y}_{j}}{4}\,$$

We indirectly estimated the power requirements of the chip during operation as the sum of the power required for the generation, transmission and post-synaptic integration of the spikes in the network^49^:

21 $${P}_{{tot}}={\sum }_{i=1}{r}_{i}\,\left(\underbrace{{E}_{{spike}}+{E}_{{enc}}}_{{spike\; generation}}\,+\,\underbrace{{N}_{{cores}}\,\left({E}_{{br}}+{E}_{{rt}}\right)}_{{spike\; transmission}}\,+\underbrace{{N}_{{post},i}{\,E}_{{pulse}}}_{{post\; syn\; spike\; int}.}\right)$$

The values and roles of the different terms are enumerated in Supplementary Tab. S2.

### Reporting summary

Further information on research design is available in the Nature Portfolio Reporting Summary linked to this article.

## Supplementary information

Supplementary Information

Description of Additional Supplementary Information

Supplementary Movie 1

Supplementary Movie 2

Supplementary Movie 3

Reporting Summary

Transparent Peer Review file

## Data Availability

The full dataset analyzed in this study is available from the corresponding author upon request. A subset of the data is openly available via Code Ocean at https://codeocean.com/capsule/1018585/tree. Moreover, source data files for all data presented in graphs within the Figures of this manuscript are provided in the following Zenodo repository: 10.5281/zenodo.18256535.

## Code availability

Reproducible code is openly available^77^ via Code Ocean at https://codeocean.com/capsule/1018585/tree.

## References

[1] Robles-De-La-Torre, G. The importance of the sense of touch in virtual and real environments. *Ieee Multimed.* **13**, 24–30 (2006).

[2] Dijkerman, H. C. & De Haan, E. H. Somatosensory processing subserving perception and action: Dissociations, interactions, and integration. *Behav. Brain Sci.* **30**, 224–230 (2007). 10.1017/S0140525X07001392 17705910

[3] Abraira, V. E. & Ginty, D. D. The sensory neurons of touch. *neuron* **79**, 618–639 (2013). 23972592 10.1016/j.neuron.2013.07.051 PMC3811145

[4] Turecek, J., Lehnert, B. P. & Ginty, D. D. The encoding of touch by somatotopically aligned dorsal column subdivisions. *Nature* **612**, 310–315 (2022). 36418401 10.1038/s41586-022-05470-x PMC9729103

[5] Vallbo, A. B. & Johansson, R. S. & others. Properties of cutaneous mechanoreceptors in the human hand related to touch sensation. *Hum. Neurobiol.* **3**, 3–14 (1984). 6330008

[6] Vallbo, A., Olausson, H., Wessberg, J. & Kakuda, N. Receptive field characteristics of tactile units with myelinated afferents in hairy skin of human subjects. *J. Physiol.* **483**, 783–795 (1995). 7776258 10.1113/jphysiol.1995.sp020622 PMC1157818

[7] Johansson, R. S. & Flanagan, J. R. Coding and use of tactile signals from the fingertips in object manipulation tasks. *Nat. Rev. Neurosci.* **10**, 345–359 (2009). 19352402 10.1038/nrn2621

[8] Mukherjee, R., Ganguly, P. & Dahiya, R. Bioinspired distributed energy in robotics and enabling technologies. *Adv. Intell. Syst.* **5**, 2100036 (2023).

[9] Kim, Y. et al. A bioinspired flexible organic artificial afferent nerve. *Science* **360**, 998–1003 (2018). 29853682 10.1126/science.aao0098

[10] Liu, F. et al. Printed synaptic transistor–based electronic skin for robots to feel and learn. *Sci. Robot.* **7**, eabl7286 (2022). 35648845 10.1126/scirobotics.abl7286

[11] Yang, J. C. et al. Electronic skin: recent progress and future prospects for skin-attachable devices for health monitoring, robotics, and prosthetics. *Adv. Mater.* **31**, 1904765 (2019). 10.1002/adma.201904765 31538370

[12] Wang, M. et al. Artificial skin perception. *Adv. Mater.* **33**, 2003014 (2021). 10.1002/adma.202003014 32930454

[13] Osborn, L. E. et al. Prosthesis with neuromorphic multilayered e-dermis perceives touch and pain. *Sci. Robot.* **3**, eaat3818 (2018). 10.1126/scirobotics.aat3818 PMC7051004 32123782

[14] Chortos, A., Liu, J. & Bao, Z. Pursuing prosthetic electronic skin. *Nat. Mater.* **15**, 937–950 (2016). 27376685 10.1038/nmat4671

[15] Bragança, S. et al.) 641–650 (Springer International Publishing, 2019) 10.1007/978-3-030-14730-3_68.

[16] Jung, B., Kim, B., Koo, J. C., Choi, H. R. & Moon, H. Joint torque sensor embedded in harmonic drive using order tracking method for robotic application. *IEEEASME Trans. Mechatron.* **22**, 1594–1599 (2017).

[17] Orekhov, A. L., Johnston, G., Abah, C., Choset, H. & Simaan, N. Towards collaborative robots with sensory awareness: preliminary results using multi-modal sensing. in *Proceedings of the IEEE ICRA workshop on Physical human-robot interaction: a design focus* 1–5 (IEEE, 2019).

[18] Kaltenbrunner, M. et al. An ultra-lightweight design for imperceptible plastic electronics. *Nature* **499**, 458–463 (2013). 23887430 10.1038/nature12314

[19] Nawrocki, R. A., Matsuhisa, N., Yokota, T. & Someya, T. 300-nm imperceptible, ultraflexible, and biocompatible e-skin fit with tactile sensors and organic transistors. *Adv. Electron. Mater.* **2**, 1500452 (2016).

[20] Xiang, S. et al. Liquid-metal-based dynamic thermoregulating and self-powered electronic skin. *Adv. Funct. Mater.* **31**, 2100940 (2021).

[21] Dahiya, R. et al. Large-area soft e-skin: the challenges beyond sensor designs. *Proc. IEEE* **107**, 2016–2033 (2019).

[22] Zhao, C., Park, J., Root, S. E. & Bao, Z. Skin-inspired soft bioelectronic materials, devices and systems. *Nat. Rev. Bioeng*. **2**, 671–690 (2024).

[23] Massari, L. et al. Functional mimicry of Ruffini receptors with fibre Bragg gratings and deep neural networks enables a bio-inspired large-area tactile-sensitive skin. *Nat. Mach. Intell.* **4**, 425–435 (2022).

[24] Taunyazov, T., Koh, H. F., Wu, Y., Cai, C. & Soh, H. Towards effective tactile identification of textures using a hybrid touch approach. in *2019**International Conference on Robotics and Automation (ICRA)* 4269–4275 (IEEE, 2019).

[25] Yan, Y. et al. Soft magnetic skin for super-resolution tactile sensing with force self-decoupling. *Sci. Robot.* **6**, eabc8801 (2021). 34043530 10.1126/scirobotics.abc8801

[26] Park, K. et al. A biomimetic elastomeric robot skin using electrical impedance and acoustic tomography for tactile sensing. *Sci. Robot.* **7**, eabm7187 (2022). 35675452 10.1126/scirobotics.abm7187

[27] Bai, N. et al. A robotic sensory system with high spatiotemporal resolution for texture recognition. *Nat. Commun.* **14**, 7121 (2023). 37963866 10.1038/s41467-023-42722-4 PMC10645869

[28] Hu, Z. et al. Machine learning for tactile perception: advancements, challenges, and opportunities. *Adv. Intell. Syst.* **5**, 2200371 (2023).

[29] Schuman, C. D. et al. Opportunities for neuromorphic computing algorithms and applications. *Nat. Comput. Sci.* **2**, 10–19 (2022). 38177712 10.1038/s43588-021-00184-y

[30] Chen, L. et al. Spike timing–based coding in neuromimetic tactile system enables dynamic object classification. *Science* **384**, 660–665 (2024). 38723082 10.1126/science.adf3708

[31] Yuan, R. et al. A calibratable sensory neuron based on epitaxial VO2 for spike-based neuromorphic multisensory system. *Nat. Commun.* **13**, 3973 (2022). 35803938 10.1038/s41467-022-31747-w PMC9270461

[32] Han, J.-K. et al. Self-powered artificial mechanoreceptor based on triboelectrification for a neuromorphic tactile system. *Adv. Sci.* **9**, 2105076 (2022). 10.1002/advs.202105076 PMC8948587 35032113

[33] Taunyazov, T., Chua, Y., Gao, R., Soh, H. & Wu, Y. Fast texture classification using tactile neural coding and spiking neural network. in *2020**IEEE/RSJ International Conference on Intelligent Robots and Systems (IROS)* 9890–9895 (IEEE, 2020).

[34] Rongala, U. B., Mazzoni, A. & Oddo, C. M. Neuromorphic artificial touch for categorization of naturalistic textures. *IEEE Trans. Neural Netw. Learn. Syst.* **28**, 819–829 (2015). 26372658 10.1109/TNNLS.2015.2472477

[35] Liu, F. et al. Neuro-inspired electronic skin for robots. *Sci. Robot.* **7**, eabl7344 (2022). 35675450 10.1126/scirobotics.abl7344

[36] Baek, E. et al. Neuromorphic dendritic network computation with silent synapses for visual motion perception. *Nat. Electron.* **7**, 454–465 (2024).

[37] Hill, K. O. & Meltz, G. Fiber Bragg grating technology fundamentals and overview. *J. Light. Technol.* **15**, 1263–1276 (1997).

[38] Pereira Resende da Costa, A. C., Filosa, M., Barbosa Soares, A. & Oddo, C. M. Type II mechanoreceptors and cuneate spiking neuronal network enable touch localization on a large-area e-skin. *Nat. Mach. Intell*. **7**, 1278–1291 (2025).

[39] Moradi, S., Qiao, N., Stefanini, F. & Indiveri, G. A scalable multicore architecture with heterogeneous memory structures for dynamic neuromorphic asynchronous processors (DYNAPs). *IEEE Trans. Biomed. Circuits Syst.* **12**, 106–122 (2018). 29377800 10.1109/TBCAS.2017.2759700

[40] Zendrikov, D., Solinas, S. & Indiveri, G. Brain-inspired methods for achieving robust computation in heterogeneous mixed-signal neuromorphic processing systems. *Neuromorphic Comput. Eng.* **3**, 034002 (2023).

[41] Wan, W. et al. A compute-in-memory chip based on resistive random-access memory. *Nature* **608**, 504–512 (2022). 35978128 10.1038/s41586-022-04992-8 PMC9385482

[42] Ambrogio, S. et al. An analog-AI chip for energy-efficient speech recognition and transcription. *Nature* **620**, 768–775 (2023). 37612392 10.1038/s41586-023-06337-5 PMC10447234

[43] Lepora, N. F. et al. Tactile superresolution and biomimetic hyperacuity. *IEEE Trans. Robot.* **31**, 605–618 (2015).

[44] Tsividis, Y. Event-driven data acquisition and digital signal processing—A tutorial. *IEEE Trans. Circuits Syst. II Express Briefs* **57**, 577–581 (2010).

[45] Rongala, U. B. et al. Intracellular dynamics in cuneate nucleus neurons support self-stabilizing learning of generalizable tactile representations. *Front. Cell. Neurosci.* **12**, 210 (2018). 30108485 10.3389/fncel.2018.00210 PMC6079306

[46] Parker, G. A. & Smith, J. M. Optimality theory in evolutionary biology. *Nature* **348**, 27–33 (1990).

[47] Jones, H. E., Andolina, I. M., Oakely, N. M., Murphy, P. C. & Sillito, A. M. Spatial summation in lateral geniculate nucleus and visual cortex. *Exp. Brain Res.* **135**, 279–284 (2000). 11131514 10.1007/s002210000574

[48] Arthur, R., Pfeiffer, R. & Suga, N. Properties of ‘two-tone inhibition’in primary auditory neurones. *J. Physiol.* **212**, 593–609 (1971). 5557062 10.1113/jphysiol.1971.sp009344 PMC1395724

[49] Zhao, J., Monforte, M., Indiveri, G., Bartolozzi, C. & Donati, E. Learning inverse kinematics using neural computational primitives on neuromorphic hardware. *npj Robot* **1**, 1 (2023).

[50] Donati, E. & Valle, G. Neuromorphic hardware for somatosensory neuroprostheses. *Nat. Commun.* **15**, 556 (2024). 38228580 10.1038/s41467-024-44723-3 PMC10791662

[51] Li, G., Liu, S., Wang, L. & Zhu, R. Skin-inspired quadruple tactile sensors integrated on a robot hand enable object recognition. *Sci. Robot.* **5**, eabc8134 (2020). 33328298 10.1126/scirobotics.abc8134

[52] Elaskar, J. et al Integrated high-speed wavelength tracking on a silicon chip. *J. Light. Technol*. **43**, 3388–3396 (2024).

[53] Euler, T., Haverkamp, S., Schubert, T. & Baden, T. Retinal bipolar cells: elementary building blocks of vision. *Nat. Rev. Neurosci.* **15**, 507–519 (2014). 25158357 10.1038/nrn3783

[54] Souffi, S., Nodal, F. R., Bajo, V. M. & Edeline, J.-M. When and how does the auditory cortex influence subcortical auditory structures? New insights about the roles of descending cortical projections. *Front. Neurosci.* **15**, 690223 (2021). 34413722 10.3389/fnins.2021.690223 PMC8369261

[55] Serre, T., Oliva, A. & Poggio, T. A feedforward architecture accounts for rapid categorization. *Proc. Natl. Acad. Sci. USA* **104**, 6424–6429 (2007). 17404214 10.1073/pnas.0700622104 PMC1847457

[56] Maheswaranathan, N. et al. Interpreting the retinal neural code for natural scenes: From computations to neurons. *Neuron* **111**, 2742–2755 (2023). 37451264 10.1016/j.neuron.2023.06.007 PMC10680974

[57] Rao, Y.-J. In-fibre Bragg grating sensors. *Meas. Sci. Technol.* **8**, 355 (1997).

[58] Werbos, P. J. Backpropagation through time: what it does and how to do it. *Proc. IEEE* **78**, 1550–1560 (1990).

[59] Wu, Y., Deng, L., Li, G., Zhu, J. & Shi, L. Spatio-Temporal Backpropagation for Training High-Performance Spiking Neural Networks. *Front. Neurosci.* **12**, 331 (2018). 29875621 10.3389/fnins.2018.00331 PMC5974215

[60] Shrestha, S. B. & Orchard, G. Slayer: spike layer error reassignment in time. *Adv. Neural Inf. Process. Syst*. **31**, 1419–1428 (2018).

[61] Eshraghian, J. K. et al. Training spiking neural networks using lessons from deep learning. *Proc. IEEE* **111**, 1016–1054 (2023).

[62] Neftci, E. O., Mostafa, H. & Zenke, F. Surrogate gradient learning in spiking neural networks: bringing the power of gradient-based optimization to spiking neural networks. *IEEE Signal Process. Mag.* **36**, 51–63 (2019).

[63] Hammouamri, I., Khalfaoui-Hassani, I. & Masquelier, T. Learning delays in spiking neural networks using dilated convolutions with learnable spacings. (ICLR, 2023).

[64] Bartolozzi, C., Indiveri, G. & Donati, E. Embodied neuromorphic intelligence. *Nat. Commun.* **13**, 1024 (2022). 35197450 10.1038/s41467-022-28487-2 PMC8866429

[65] Rathi, N. et al. Exploring neuromorphic computing based on spiking neural networks: algorithms to hardware. *ACM Comput. Surv.* **55**, 1–49 (2023).

[66] Mayr, C., Hoeppner, S. & Furber, S. Spinnaker 2: a 10 million core processor system for brain simulation and machine learning. *ArXiv Prepr*. *ArXiv191102385* (2019).

[67] Pei, J. et al. Towards artificial general intelligence with hybrid Tianjic chip architecture. *Nature* **572**, 106–111 (2019). 31367028 10.1038/s41586-019-1424-8

[68] Orchard, G. et al. Efficient neuromorphic signal processing with loihi 2. in *2021 IEEE Workshop on Signal Processing Systems (SiPS)* 254–259 (IEEE, 2021).

[69] Pehle, C. et al. The BrainScaleS-2 accelerated neuromorphic system with hybrid plasticity. *Front. Neurosci.* **16**, 795876 (2022). 35281488 10.3389/fnins.2022.795876 PMC8907969

[70] Yao, M. et al. Spike-based dynamic computing with asynchronous sensing-computing neuromorphic chip. *Nat. Commun.* **15**, 4464 (2024). 38796464 10.1038/s41467-024-47811-6 PMC11127998

[71] Frenkel, C., Bol, D. & Indiveri, G. Bottom-up and top-down approaches for the design of neuromorphic processing systems: Tradeoffs and synergies between natural and artificial intelligence. *Proceedings of the IEEE* **111**, 623–652 (2023).

[72] Mead, C. Neuromorphic electronic systems. *Proc. IEEE* **78**, 1629–1636 (1990).

[73] Mead, C. How we created neuromorphic engineering. *Nat. Electron.* **3**, 434–435 (2020).

[74] Indiveri, G. et al. Neuromorphic silicon neuron circuits. *Front. Neurosci.* **5**, 73 (2011). 21747754 10.3389/fnins.2011.00073 PMC3130465

[75] Chicca, E., Stefanini, F., Bartolozzi, C. & Indiveri, G. Neuromorphic electronic circuits for building autonomous cognitive systems. *Proc. IEEE* **102**, 1367–1388 (2014).

[76] Brette, R. & Gerstner, W. Adaptive exponential integrate-and-fire model as an effective description of neuronal activity. *J. Neurophysiol.* **94**, 3637–3642 (2005). 16014787 10.1152/jn.00686.2005

[77] Ortone, A. et al. Code Ocean capsule, Bioinspired spiking architecture enables energy constrained touch encoding (2025). 10.1038/s41467-026-68858-7 PMC12953599 41605933

