# Supplementary material for: Bioinspired spiking architecture enables energy constrained touch encoding
Source: Nat Commun. 2026 Jan 28;17:2108. doi: 10.1038/s41467-026-68858-7 (PMC12953599; doi:10.1038/s41467-026-68858-7)
Supplement: Supplementary file 1 — Supplementary Information [file 41467_2026_68858_MOESM1_ESM.pdf]

Supplementary Information for

Bioinspired spiking architecture enables energy constrained touch encoding

Andrea Ortone, Mariangela Filosa, Giacomo Indiveri, Giuseppe Desoli, Alberto Mazzoni, Calogero Maria Oddo*

*Corresponding author: calogero.oddo@santannapisa.it

Contents

1 Supplementary Figures **2**

2 Supplementary Tables **8**

3 Supplementary Methods **9**

3.1 FI curve of the employed neurons 9

3.2 Preprocessing of the input signals to the SNN 9

3.3 Learning procedure 11

3.4 Weight quantization 14

3.5 Reproduction of localization method in[1] 14

3.6 Details on DYNAP-SE implementation 15

3.6.1 Synapses selection 15

3.6.2 Training with chip in-the-loop 15

3.6.3 Optimized neuron association 17

3.7 Summary of the employed parameters 19

4 Supplementary Results **20**

4.1 Scalability to larger robotic surfaces and wiring complexity 20

Supplementary References **21**

Other Supplementary Materials for this manuscript include the following:

- Supplementary Movies S1 to S3.

1 Supplementary Figures

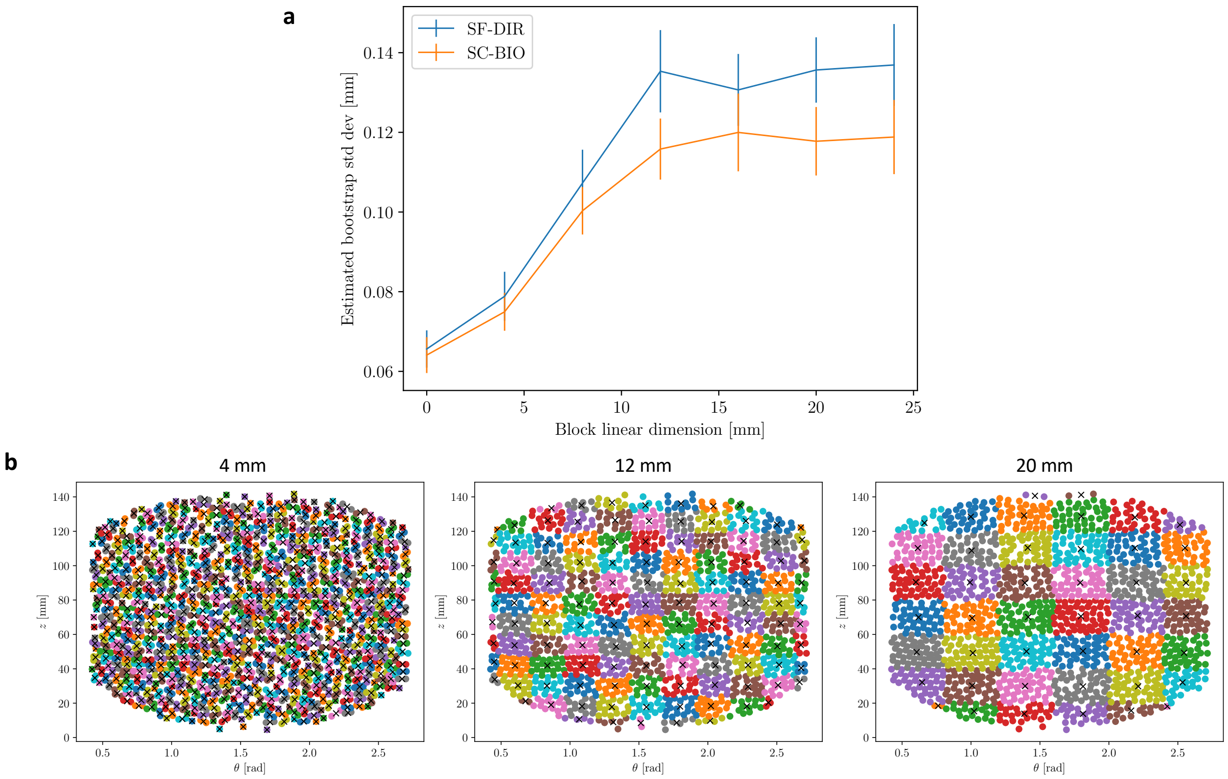

Supplementary Fig. S1: **Implementation of blocked bootstrap for estimating the standard deviation of the localization error.** (**a**) Standard deviation of the median applying blocked resampling with replacement, repeated 100 times (internal loop), as a function of block sizes and for the two considered architectures. Central values and error bars represent means and standard deviations, computed over 40 repetitions (external loop). The estimated standard deviation of the localization error increases until it reaches a plateau, when the block-size exceeds ≈ 12 mm. (**b**) Considered blocks in (a) for some values of block-size.

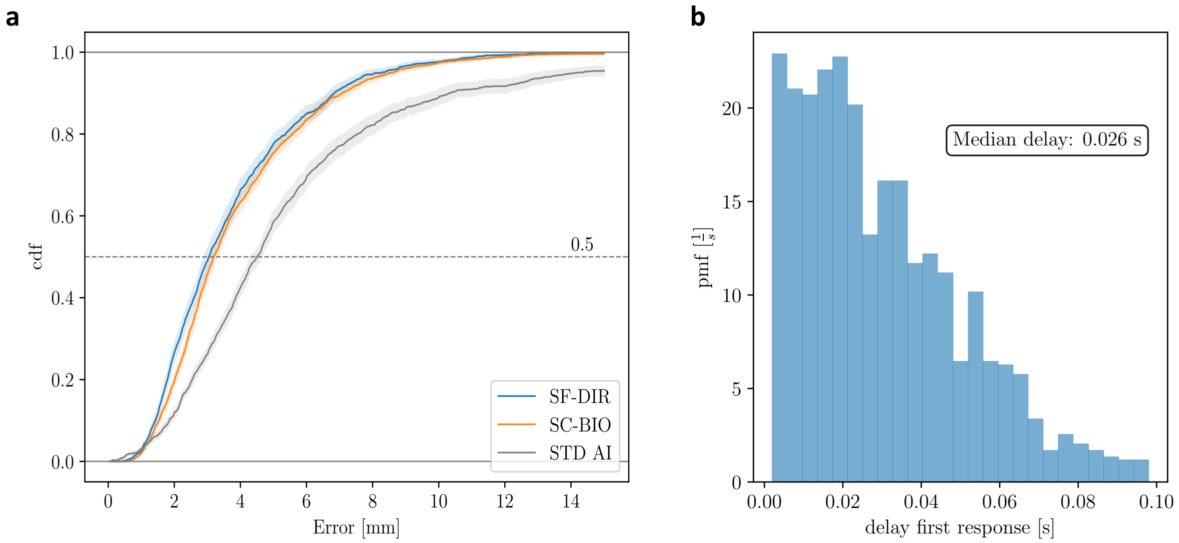

Supplementary Fig. S2: **Detection and localization performance of the tactile system.** (**a**) Cumulative distribution function of the performed localization error for the considered decoding strategies (blue: SF-DIR architecture; orange: SC-BIO architecture; gray: replication of the strategy described in [23] and based on standard paradigms of AI). Shaded regions represent standard deviations of the median error computed via blocked bootstrap. (**b**) Histogram of the network detection delays for the SC-DIR network.

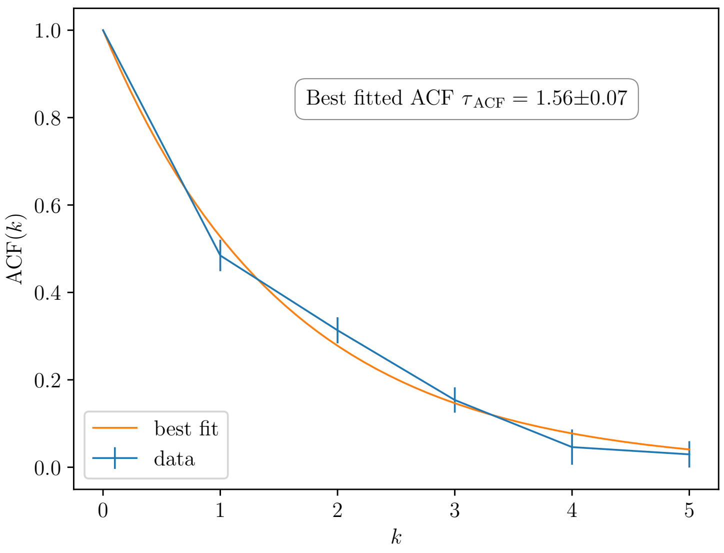

Supplementary Fig. S3: **Autocorrelation function of the median localization error among different degrees of weight quantization.** Central values and error bars represent the means and standard deviations computed across different numbers of output neurons N_{output}. Best fit is performed using a decreasing exponential function $\mathrm{ACF}(k) = \exp(-k/\tau_{\mathrm{ACF}})$. GOF has been estimated through χ^2 test and resulted in $p_{\mathrm{value}} = 0.78$.

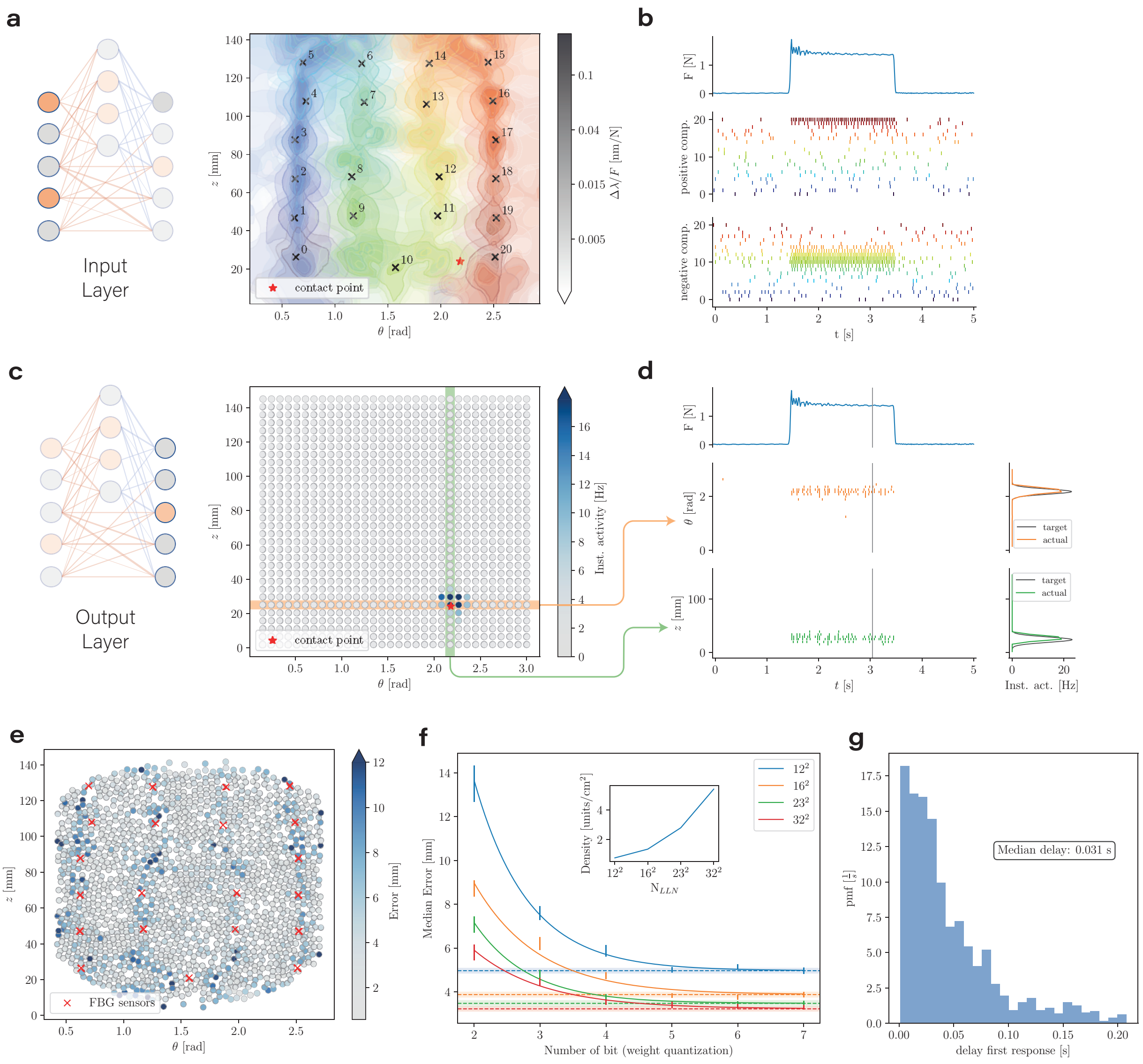

Supplementary Fig. S4: **Localization and detection performance for the SC-BIO architecture in unconstrained conditions.** (**a**) Contour plot of the intensity of the response of the 21 FBG sensors as a function of the position of the indentation showing that the sensitive regions of the sensors are overlapped. (**b**) Evolution through time of the force applied over the surface of the e-skin during indentation (top row) and spike trains of the input layer neurons associated with the positive (middle row) and negative (bottom row) components of the FBG signals. (**c**) Instantaneous activity of the neurons in the output layer when an indentation is performed. (**d**) Evolution through time of the applied force during an indentation (top row) and spike trains of the output layer neurons highlighted in subplot (c). The estimated position of the indentation is computed as the barycentre of the activities of the output neurons. (**e**) Scatter plot of the localization error over the surface of the skin. (**f**) Median localization error for different degrees of weight quantization and different numbers of output neurons. (**g**) Histogram of the network detection delays.

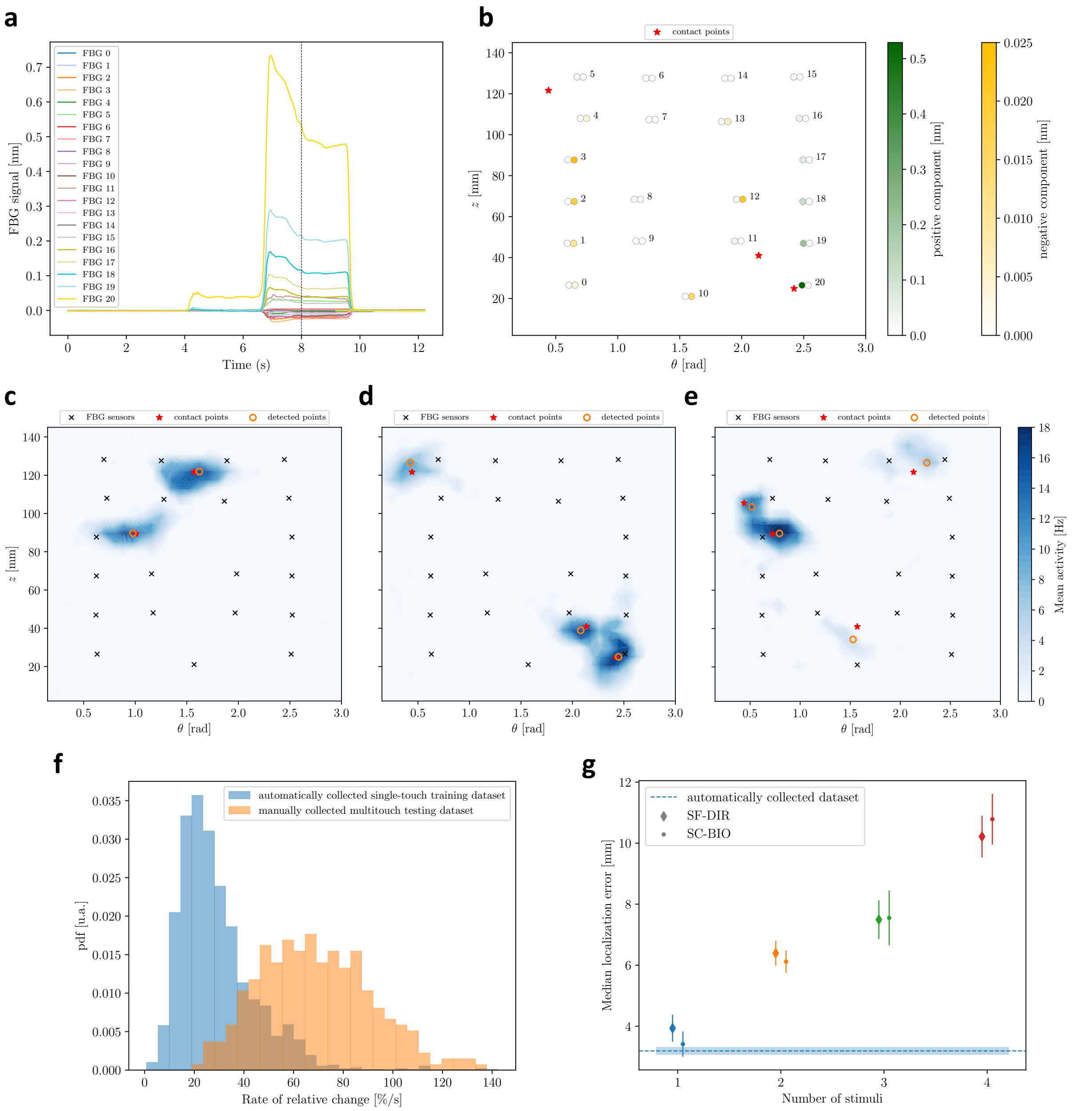

Supplementary Fig. S5: **Tactile decoding under multitouch and dynamic conditions.** (**a**) Temporal evolution of the 21 FBG signals in response to a representative triple contact trial. (**b**) Positive and negative components of the FBG signals at t = 8 s (highlighted in panel (a)), with the target positions of contact points (red stars). (**c-e**) Mean spiking activity of the neurons in the output layer, with the target positions of contact points (red stars) and detected contact points (orange unfilled circles) under double (c), triple (d) and quadruple (e) contact trials. Note that panel (d) is associated with input signals shown in panels (a) and (b). (**f**) Distributions of the rate of relative change in the automatically collected dataset used for training and in the manually collected dataset employed for testing generalization abilities to multitouch and more dynamic tactile interactions. (**g**) Median Localization error under multitouch and more dynamic conditions for the bioinspired (SC-BIO) and alternative (SF-DIR) architectures. The dashed horizontal line indicates the median localization error obtained in the automatically collected evaluation dataset. Central markers denote median localization errors, error-bars represent standard deviations estimated via bootstrap techniques.

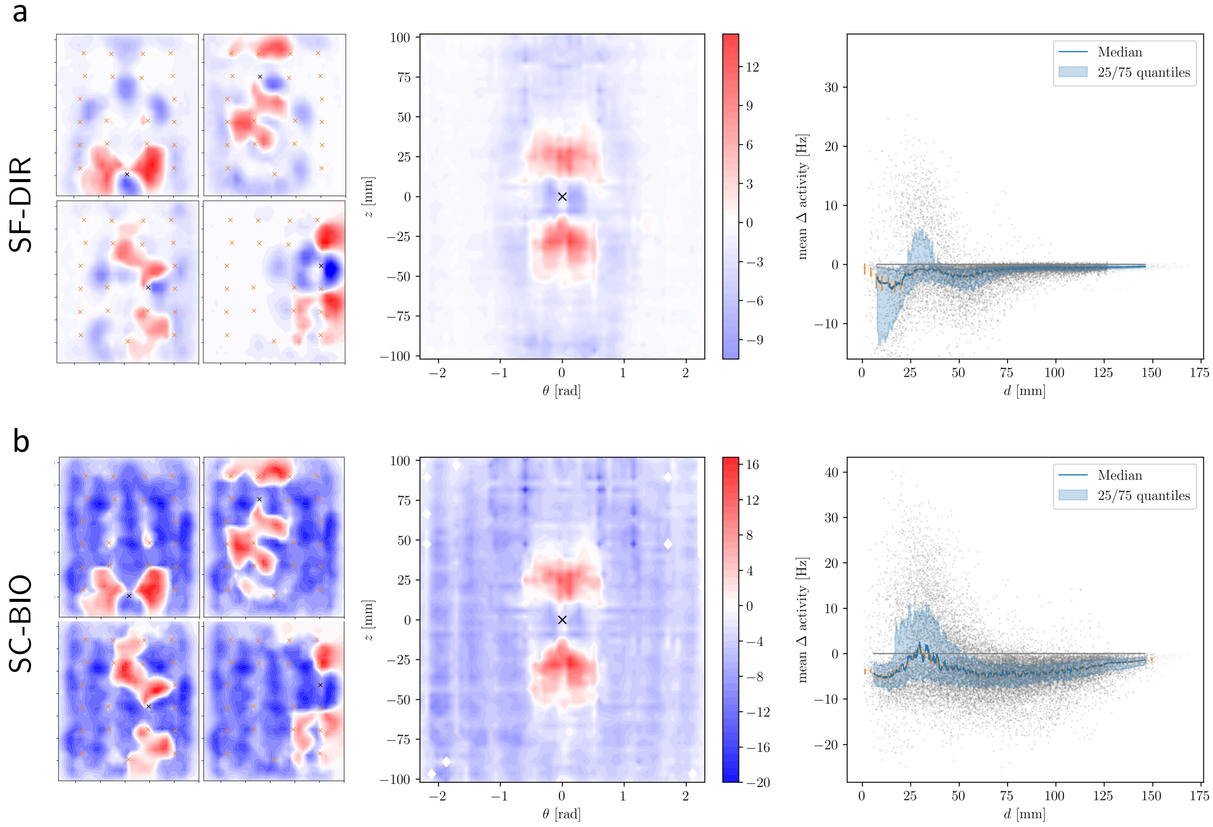

Supplementary Fig. S6: **Emergent functional connectivity properties between input neurons mimicking SA2 mechanoreceptors (negative component of $\Delta\lambda$) and the output layer**. (**a-b**) Emergent functional connectivity for the SF-DIR and SC-BIO architectures respectively. (left) Connectivity patterns from 4 individual input neurons (black cross) and output neurons; (center) connectivity patterns averaged across input neurons mimicking SA2 mechanoreceptors; (right) projection over the radial dimension of the connectivity properties shown in the central panels: each dot represents a pair (input, output) neuron: the continuous blue-line and the shaded regions represent the median and interquartile values, respectively.

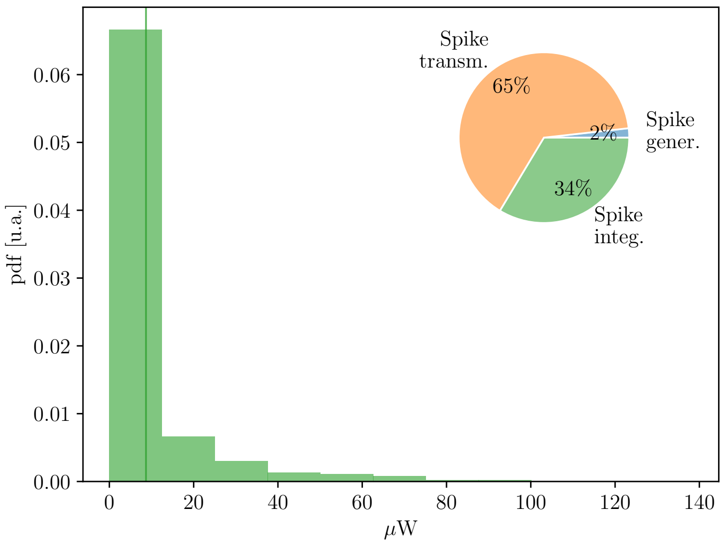

Supplementary Fig. S7: **Estimated power consumption of the DYNAP-SE chip in resting conditions.** Histogram of the power consumption of the network in resting conditions and relative contribution of spike generation, transmission and post-synaptic integration mechanisms to the total power consumption of the chip.

2 Supplementary Tables

$N_{\mathbf{output}}$ **(architecture)**	a	$\tau_{\mathbf{n\_bit}}$	**GOF** p**-value**
12^2 (SF-DIR)	27 ± 7	1.0 ± 0.1	0.21
16^2 (SF-DIR)	30 ± 4	0.88 ± 0.05	0.88
23^2 (SF-DIR)	20 ± 2	0.94 ± 0.04	0.53
32^2 (SF-DIR)	19 ± 5	0.90 ± 0.09	0.91
12^2 (SC-BIO)	100 ± 32	0.81 ± 0.09	0.13
16^2 (SC-BIO)	39 ± 20	0.97 ± 0.28	0.001
23^2 (SC-BIO)	41 ± 13	0.83 ± 0.10	0.38
32^2 (SC-BIO)	17 ± 7	1.06 ± 0.18	0.22

Supplementary Tab. S1: Best fitted parameters and goodness-of-fit (GOF) values for the exponential model describing the median localization error as a function of different degrees of weight quantization (see Fig. 3g and Methods in the Main Text).

**Symbol**	**Operation**	**Energy requirement [pJ]**
E_{spike}	Generate one spike	883
E_{enc}	Encode one spike and append destinations	883
E_{br}	Broadcast events to the same core	6840
E_{rt}	Route events to a different core	360
E_{pulse}	Extend generated pulse	324

Supplementary Tab. S2: Energy requirement for the generation, transmission, and post-synaptic integration of spikes in the DYNAP-SE processor.

3 Supplementary Methods

3.1 FI curve of the employed neurons

The FI curve of the neurons with the adopted neural parameters is plotted in Supplementary Fig. S8. Note that the introduction of the external poissonian input makes the FI curve more regular in the regime of transition between active and silent states ($I_0 \sim 15\,\text{pA}$).

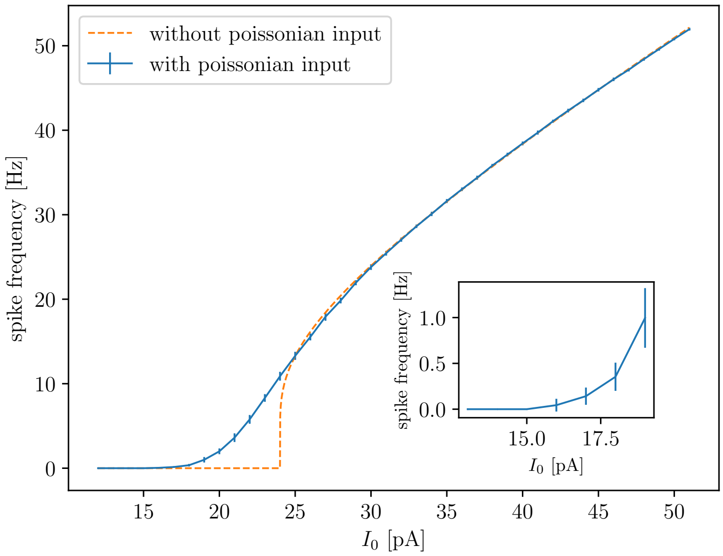

Supplementary Fig. S8: FI curve of the adopted neuron model with (blue line) and without (orange dashed line) poissonian input. Error bars indicate the standard deviation of output frequencies in simulations with duration 12 s. For the non-noisy condition, the theoretical expected value $\left(t_{ref} - \frac{C_m}{g_L} \ln\left(1 - \frac{g_L}{I_0}(V_{th} - V_{res})\right)\right)^{-1}$ is plotted.

3.2 Preprocessing of the input signals to the SNN

The FBG signals $\{\Delta\lambda_i(t)\}_{i=1,n_{\text{FBG}}}$ represent the time-varying shifts of the reflected wavelength of each sensor embedded in the skin. These shifts can be positive or negative, depending on the nature of the stimulus and the relative position between the sensor and the performed stimulus (see Figure 1 in the main text and Supplementary Fig. S9). The two components of each FBG sensor are separated, transformed through non-linear functions $f^{(\cdot)}$ and finally fed as external currents into two different neurons of the input layer:

$$\forall i \quad \Delta\lambda_i(t) \quad \longmapsto \quad \begin{cases} \Delta\lambda_i^{(+)}(t) = \texttt{ReLU}\left(\Delta\lambda_i(t)\right) \\ \Delta\lambda_i^{(-)}(t) = \texttt{ReLU}\left(-\Delta\lambda_i(t)\right) \end{cases} \quad \longmapsto \quad \begin{cases} I_i^{(+)}(t) & = f^{(+)}\left(\Delta\lambda_i^{(+)}(t); \text{LF}\right) \\ I_i^{(-)}(t) & = f^{(-)}\left(\Delta\lambda_i^{(-)}(t); \text{LF}\right) \end{cases}$$

The function $f^{(\cdot)}$ is defined as:

$$f^{(\cdot)}(x; a, b, c) = a \log(1 + bx) + c \tag{s1}$$

where the a, b and c parameters have been chosen separately for the two components to ensure that:

- *small* signals $\Delta\lambda_i^{(\cdot)}(t)$ can generate output spikes, which implies $c^{(+)} = c^{(-)} \sim 15\,\text{pA}$ (see FI curve in Supplementary Fig. S8);

- *large* signals $\Delta\lambda_i^{(\cdot)}(t)$ can generate *high* output firing rate. Specifically, we consider a constraint point $(x_Q^{(\cdot)}, y_Q^{(\cdot)})$ and set a and b so that $f(x_Q^{(\cdot)}; a, b, c) = y_Q^{(\cdot)}$. The coordinates $(x_Q^{(\cdot)}, y_Q^{(\cdot)})$ have been fixed to:

$$x_Q^{(\cdot)} = 99\%\ \texttt{quantile}\text{ of the non-null values of }\Delta\lambda_i^{(\cdot)} \qquad y_Q^{(\cdot)} = 50\,\text{pA}$$

  The value of $y_Q^{(\cdot)}$ implies an output firing rate of $\sim 50\,\text{Hz}$ (see Supplementary Fig. S8) which is far from the saturation value $\sim 1/t_{ref}$;

- the degree of non linearity of the transformation (which is a function of a and b) is explicit and controlled by the LF parameter as follows:

$$\text{LF} = \frac{\frac{\text{d}}{\text{d}x}\left[f^{(\cdot)}(x; a, b, c)\right]}{m(x_Q^{(\cdot)}, y_Q^{(\cdot)}, c)}\Bigg|_{x=0} - 1 \qquad \text{with} \qquad m(x_Q^{(\cdot)}, y_Q^{(\cdot)}, c) = \frac{y_Q^{(\cdot)} - c}{x_Q^{(\cdot)}} \tag{s2}$$

  which means: the slope of $f^{(\cdot)}(x; a, b, c)$ in the origin is LF + 1 times the slope of the straight line connecting the points $(0, c)$ and $(x_Q^{(\cdot)}, y_Q^{(\cdot)})$. As a consequence, LF = 0 implies a linear transformation and LF $\gg 1$ implies a large degree of non-linearity (see Supplementary Fig. S10).

Note also that, since the baseline of the external current is trained for each neuron (see Section 3.3), the choice of $c^{(\cdot)}$ represents only an initial guess.

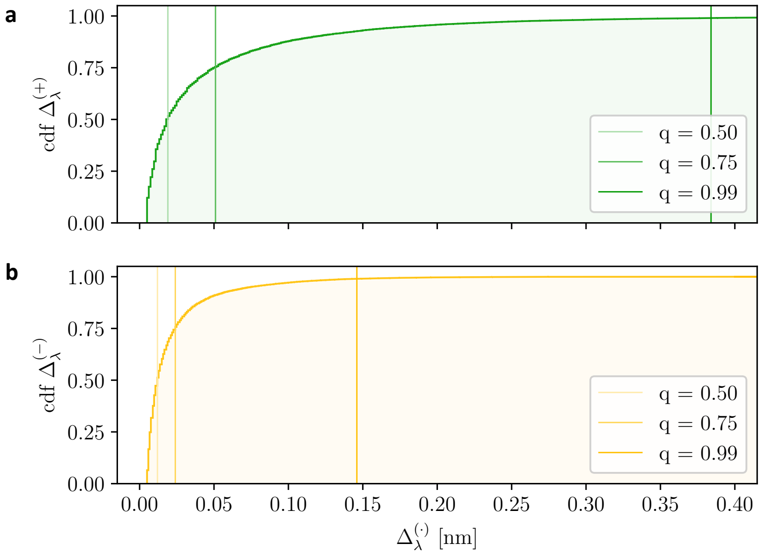

Supplementary Fig. S9: Cumulative distribution function of the positive (a) and negative (b) components of $\Delta\lambda$. Vertical lines highlight the 0.5, 0.75 and 0.99 quantiles of the distribution quantified in Supplementary Tab. S3.

Quantile	$\Delta\lambda^{(+)}$ [nm]	$\Delta\lambda^{(-)}$ [nm]
0.5	0.019	0.012
0.75	0.051	0.024
0.99	0.384	0.146

Supplementary Tab. S3: Quantiles of the positive and negative components of $\Delta\lambda$.

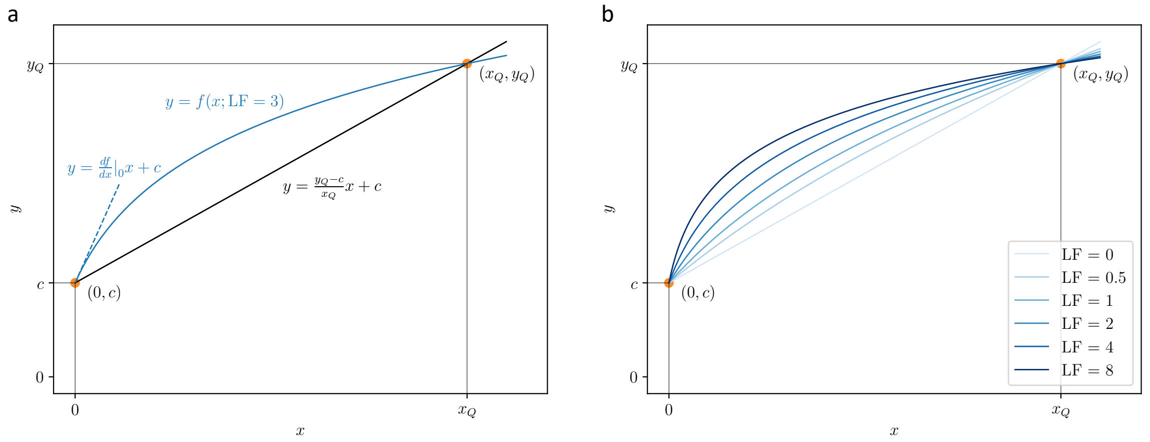

Supplementary Fig. S10: Graph of the transformation $f^{(\cdot)}$ applied to the two components of the FBG signals. (**a**) Effects of the parameters c, x_Q and y_Q on the transformation. (**b**) Impact of the parameter LF on the degree of non-linearity of the transformation.

3.3 Learning procedure

Recent advancements in the training of SNN leverage backpropagation through time[2] and surrogate gradients[3;4;5] to address the non-differentiability of the spike mechanism. While these techniques achieve strong results on both standard and neuromorphic datasets[6;7], their computational complexity and non-local operations raise doubts on their biological plausibility. Conversely, purely biomimetic methods like STDP[8] and BCM[9] learning are traditionally unsupervised and struggle with complex tasks, suggesting that the brain employs additional mechanisms. Our learning procedure balances these extremes by using error feedback from deeper layers without needing to store neuron states or exact spike times during the forward pass. Although the biological plausibility of backpropagation is debated[10], recent studies suggest that some of its simplified and approximated versions lead to weight updates that are coherent with backpropagation[11;12].

In this section, we describe the learning algorithm we employed in this work. Given:

$$\mathtt{W} \in \mathbb{R}^+ \qquad t \in \mathbb{R} \qquad l \in [1, L] \qquad j \in \left[1, N^{(l)}\right] \tag{s3}$$

we define:

- the *instantaneous* activity $y_j^{(l)}(t)$ as the number of spikes emitted by the jth neuron in layer l in the time interval $[t - \mathtt{W}/2, t + \mathtt{W}/2]$;
- the *instantaneous* error $E_i(t) = E\left(y_i^a(t), y_i^d(t)\right)$ as a function which measures the performed error at time t given the *instantaneous* actual activity $y_i^{(L)}(t)$ of the ith output neuron and the related target output $y_i^*(t)$ (see Methods).

The adopted training algorithm updates the trainable parameters every `ind_per_batch` indentations according to:

$$\Delta w_{ji}^{(l-1)} = \sum_{k=1}^{\mathtt{N_S}} \mathrm{d}w_{ji}^{(l-1)}(k\mathtt{t_S}) \qquad \Delta I_{0,j}^{(l)} = \sum_{k=1}^{\mathtt{N_S}} \mathrm{d}I_{0,j}^{(l)}(k\mathtt{t_S}) \tag{s4}$$

$$\mathrm{d}w_{ji}^{(l-1)}(t) = -\lambda \cdot m\left(I_j^{(l)}(t)\right) H\left(y_j^{(l)}(t)\right) \tau_{ji}^{(l)} y_i^{(l-1)}(t) \tilde{E}_j^{(l)}(t) \tag{s5}$$

$$\mathrm{d}I_{0,j}^{(l)}(t) = -\lambda \cdot m\left(I_j^{(l)}(t)\right) H\left(y_j^{(l)}(t)\right) \mathtt{W} \tilde{E}_j^{(l)}(t) \tag{s6}$$

where:

- $\mathtt{t_S} \in \mathbb{R}^+$ is a positive time constant setting the stride with which the *instantaneous* error $E(t)$ is evaluated during training;
- $\mathtt{N_S} \in \mathbb{N}$ is the number of times that the error is evaluated within each batch:

$$\mathtt{N_S} = \lfloor \frac{\mathtt{ind\_per\_batch} \cdot \mathtt{ind\_duration}}{\mathtt{t_S}} \rfloor$$

- $\lambda \in \mathbb{R}^+$ is the learning rate;
- $m \in \mathbb{R}^+$ is the slope of the FI curve in the operation point:

$$m\left(I_j^{(l)}(t)\right) = \frac{\mathrm{dF}}{\mathrm{d}I}\Big|_{I_j^{(l)}(t)}$$

  During the training procedure, the FI curve is approximated as a $\mathtt{ReLU}$ function and the value of $m = m_0$ is fixed to $m_0 \sim \frac{0.02\,\mathrm{kHz}}{10\,\mathrm{pA}}$ in the region where $\mathrm{F}(I) > 0$ (see Supplementary Fig. S8);
- $\tau_{ji}^{(l)} \in \mathbb{R}^+$ is the characteristic decay time of $I_j^{(l)}(t)$: hence $\tau_{ji}^{(l)} = \tau_{ex}$ if $w_{ji}^{(l-1)} > 0$ else $\tau_{ji}^{(l)} = \tau_{in}$;
- $H(x)$ is the Heaviside step function ($H(x) = 1$ if $x > 0$ and $H(x) = 0$ otherwise);
- $\tilde{E}_j^{(l)}(t)$ is the *generalized instantaneous* error of the j-th neuron in layer l and is defined as:

$$\tilde{E}_j^{(l)}(t) = \begin{cases} \displaystyle\sum_{k=1}^{N^{(l+1)}} m_0 H\left(y_k^{(l+1)}(t)\right) \tau_{kj}^{(l+1)} w_{kj}^{(l)} \tilde{E}_k^{(l+1)}(t) & \text{if } l < L \\ G\left(y_j^{(L)}(t), y_j^*(t)\right) := \dfrac{\partial E\left(y_j^{(L)}(t), y_j^*(t)\right)}{\partial y_j^L(t)} & \text{otherwise} \end{cases}$$

The function G is defined as:

$$G\left(y_j^{(L)}(t), y_j^*(t)\right) = \begin{cases} 0 & \text{if } y_j^{(L)}(t) = \mathtt{Median}_{\mathrm{POISS}}(\mu = y_j^*) \\ \mathtt{cdf}_{\mathrm{POISS}}\left(y_j^{(L)}(t); \mu = y_j^*(t)\right) - \dfrac{1}{2} & \text{otherwise} \end{cases}$$

and plotted in Supplementary Fig. S11. The target output y_j^* of each neuron in the output layer is shaped by a Gaussian kernel centred at the contact point of the presented stimulus.

The described learning rule results from the principle of error-minimization, by applying the stochastic gradient descent algorithm:

$$\mathrm{d}w_{ji}^{(l-1)}(t) = -\lambda \frac{\partial E(t)}{\partial w_{ji}^{(l-1)}} \qquad \mathrm{d}I_{0,j}^{(l)}(t) = -\lambda \frac{\partial E(t)}{\partial I_{0,j}^{(l)}}$$

Let us consider a time t and the related time window $[t - \mathtt{W}/2, t + \mathtt{W}/2]$. The computational graph of the forward pass for the instantaneous activity $y_j^{(l)}(t)$ is given by:

$$y_i^{(l-1)}(t) \xmapsto{w_{ji}^{(l-1)}} I_j^{(l)}(t) = I_{ex,j}^{(l)} + I_{in,j}^{(l)} + I_{0,j}^{(l)} + I_{ext,j}^{(l)} \xmapsto{\text{FI curve}} F_j^{(l)}(t) \xmapsto{\cdot/\mathtt{W}} y_j^{(l)}(t)$$

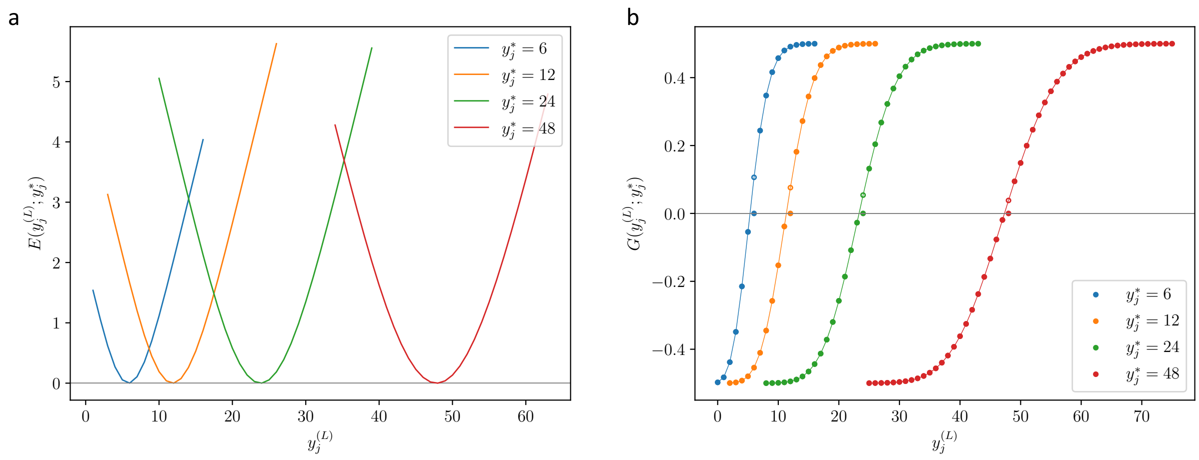

Supplementary Fig. S11: (**a-b**) Graph of the error function $E\left(y_j^{(L)}, y_j^*\right)$ and of its derivative $G\left(y_j^{(L)}, y_j^*\right)$ for different values of y_j^*.

Hence, applying the chain rule:

$$\frac{\partial E(t)}{\partial w_{ji}^{(l-1)}} = \frac{\partial E(t)}{\partial y_j^{(l)}(t)} \frac{\partial y_j^{(l)}(t)}{\partial w_{ji}^{(l-1)}} \tag{s7}$$

$$\frac{\partial y_j^{(l)}(t)}{\partial w_{ji}^{(l-1)}} = \frac{\partial y_j^{(l)}(t)}{\partial F_j^{(l)}(t)} \frac{\partial F_j^{(l)}(t)}{\partial w_{ji}^{(l-1)}} = \mathtt{W} \frac{\partial F_j^{(l)}(t)}{\partial I_j^{(l)}(t)} \frac{\partial I_j^{(l)}(t)}{\partial w_{ji}^{(l-1)}} \tag{s8}$$

$$\frac{\partial E(t)}{\partial y_j^{(l)}(t)} \overset{l \leq L}{=} \tilde{E}_j^{(l)} = \frac{\partial E(t)}{\partial y_k^{(l+1)}(t)} \frac{\partial y_k^{(l+1)}(t)}{\partial y_j^{(l)}(t)} = \tilde{E}_k^{(l+1)} \frac{\partial F_k^{(l+1)}(t)}{\partial F_j^{(l)}(t)} = \tilde{E}_k^{(l+1)} \frac{\partial F_k^{(l+1)}(t)}{\partial I_k^{(l+1)}(t)} \frac{\partial I_k^{(l+1)}(t)}{\partial F_j^{(l)}(t)} \tag{s9}$$

In order to compute the terms $\frac{\partial I_j^{(l)}(t)}{\partial w_{ji}^{(l-1)}}$ and $\frac{\partial I_k^{(l+1)}(t)}{\partial F_j^{(l)}(t)}$ the fluctuations of the currents I are neglected:

$$\begin{aligned} I_j^{(l)}(t) &= w_{ji}^{(l-1)} \left(e^{-x/\tau_{ji}^{(l)}} H(x) * \sum_{t_i} \delta(x - t_i) \right)(t) \approx w_{ji}^{(l-1)} \, \mathbb{E}\left[e^{-x/\tau_{ji}^{(l)}} H(x) * \sum_{t_i} \delta(x - t_i)(t) \right] \\ &= w_{ji}^{(l-1)} F_i^{(l-1)}(t) \tau_{ji}^{(l)} \end{aligned}$$

which leads to:

$$\frac{\partial y_j^{(l)}(t)}{\partial w_{ji}^{(l-1)}} = \mathtt{W} \frac{\partial F_j^{(l)}(t)}{\partial I_j^{(l)}(t)} F_i^{(l-1)}(t) \tau_{ji}^{(l)} = \frac{\partial F_j^{(l)}(t)}{\partial I_j^{(l)}(t)} y_i^{(l-1)}(t) \tau_{ji}^{(l)}$$

$$\frac{\partial E(t)}{\partial y_j^{(l)}(t)} \overset{l \leq L}{=} \tilde{E}_j^{(l)} = \tilde{E}_k^{(l+1)} \frac{\partial F_k^{(l+1)}(t)}{\partial I_k^{(l+1)}(t)} w_{kj}^{(l)} \tau_{kj}^{(l+1)}$$

Finally, we write the terms $\frac{\partial F_j^{(l)}(t)}{\partial I_j^{(l)}(t)}$ as:

$$\frac{\partial F_j^{(l)}(t)}{\partial I_j^{(l)}(t)} = m\left(I_j^{(l)}(t)\right) H\left(I_j^{(l)}(t) - \bar{I}_j^{(l)}\right) \approx m\left(I_j^{(l)}(t)\right) H\left(y_j^{(l)}(t)\right)$$

which leads to equation (s5). The same computations and approximations lead to equation (s6).

3.4 Weight quantization

Given the distribution $p(w)$ of the post-training weights and the number of available bit $n_{\texttt{bit}}$, the weights have been quantized according to (see Supplementary Fig. S12):

$$w_Q(w) = \begin{cases} \texttt{round}\left(\frac{w}{\Delta w^+}\right)\Delta w^+ & \text{if } w > 0 \\ \texttt{round}\left(\frac{w}{\Delta w^-}\right)\Delta w^- & \text{otherwise} \end{cases} \tag{s10}$$

where Δw^+ and Δw^- are given by:

$$\Delta w^+ = \frac{w_M}{2^{n_{\texttt{bit}}-1}} \qquad w_M = 1 - \frac{1}{(1+2^{n_{\texttt{bit}}-1})^2} \ \texttt{quantile} \text{ of the positive weights distribution}$$

$$\Delta w^- = \frac{-w_m}{2^{n_{\texttt{bit}}-1}} \qquad w_m = \frac{1}{(1+2^{n_{\texttt{bit}}-1})^2} \ \texttt{quantile} \text{ of the negative weights distribution}$$

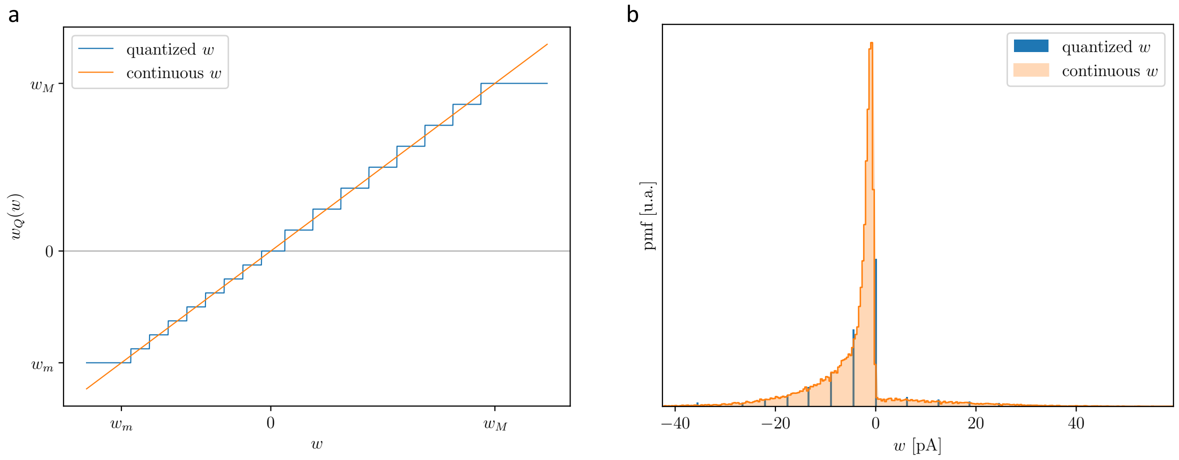

Supplementary Fig. S12: Illustration of the employed rule of quantization. (**a**) Effects of the parameters w_m and w_M on the quantization rule in the case of $n_{\texttt{bit}} = 4$. (**b**) Distribution of the post-training weights between input and output neurons for the SF-DIR architecture before and after quantization.

3.5 Reproduction of localization method in [1]

In order to fairly compare our results with those in [1], we reproduced the same localization strategy on our dataset. In particular, to resolve the localization problem, four neural networks were trained using four distinct grid configurations as target classes. These virtual grids comprised 48, 54, 56, and 61 squares, each measuring 20 mm × 20 mm. The grid with 48 squares (8 × 6) served as a reference, while the remaining grids were shifted vertically (VSG), horizontally (HSG), and diagonally (DSG) by half a square. Each neural network was trained independently to assign a weight and thus a classification percentage to each square of its respective grid. To achieve finer resolution, the outcomes of the four networks were then combined. As in [1], the errors are computed in the bidimensional projection space (x, z) and only indentations with $\theta \in [-\pi/2, \pi/2]$ are considered.

Each classification network featured three hidden layers utilizing the `ReLU` activation function, a dropout layer to prevent overfitting, and an output layer employing the `softmax` activation function.

Since our ROI region is larger than the one considered in [1], we enlarged the reference grid from 6×5 to 8×6 and increased the number of neurons in each of the hidden layers from $[100, 100, 50]$ to $[120, 120, 60]$.

3.6 Details on DYNAP-SE implementation

Analog neurons in the DYNAP-SE processor integrate an exponential LIF neural dynamics and exhibit neuron-to-neuron heterogeneity[13] when subjected to constant external input currents or excitatory and inhibitory input spike trains (see Supplementary Fig. S13). To adapt our 2-bit quantized network to the DYNAP-SE processor, tackling and exploiting this variability, we pursued the following strategy:

- we coupled each of the four 2bit-quantized weights $\{W^-, w^-, w^+, W^+\}$ to a synapse of the neuromorphic chip (see Section 3.6.1);
- for each considered core and for each k-fold validation split, we employed the training dataset to tune the shared values of the weights and the baseline current of the neurons to minimize the mismatch between the hardware and simulated neurons (see Section 3.6.2);
- we employed the training dataset to optimize the process of association between hardware and simulated neurons (see Section 3.6.3);

Eventually, we employed the testing split to evaluate the localization performance of the hardware implementation of the tactile system. Since the processor does not support time varying currents in input to the analog neurons and because the majority of neurons in the energy efficient SC-BIO architecture (1024 out of 1072 $\sim$ 95%) are located in the output layer, only the output neurons have been implemented in the DYNAPSE chip.

3.6.1 Synapses selection

Each neuron in the DYNAP-SE processor is integrated with 4 synapses: 2 are excitatory (`AMPA` and `NMDA`) and 2 are inhibitory (`GABA_A` and `GABA_B`). The inhibitory synapse `GABA_B` integrates shunting inhibition and is therefore not employed. In order to have two levels of inhibitory weights, the stronger weight W^- is implemented employing the same synapse of w^- (`GABA_A`) and duplicating each input spike (see Supplementary Tab. S4).

Weight	Employed synapse	Spike replication factor
W^-	`GABA_A`	2
w^-	`GABA_A`	1
w^+	`AMPA`	1
W^+	`NMDA`	1

Supplementary Tab. S4: Strategy of implementation of the 2bit-quantized weights on the DYNAP-SE processor.

3.6.2 Training with chip in-the-loop

The values of the weights associated to the `AMPA`, `NMDA` and `GABA_A` synapses and the value of the baseline input current have been tuned so that the activities of the neurons in the chip best match those of the simulated neurons. To do so, we built a dataset consisting of pairs $(s, \mathtt{n})$ where s is one of the stimuli in the training set and `n` is an output neuron of the simulated network. For each of these pairs, we set up the specific connectivity between the input neurons and the selected output neuron `n` for *all* neurons of the DYNAP-SE chip. We then minimized the discrepancy between the median activity of the hardware neurons (DYN-act) and the activity of the simulated

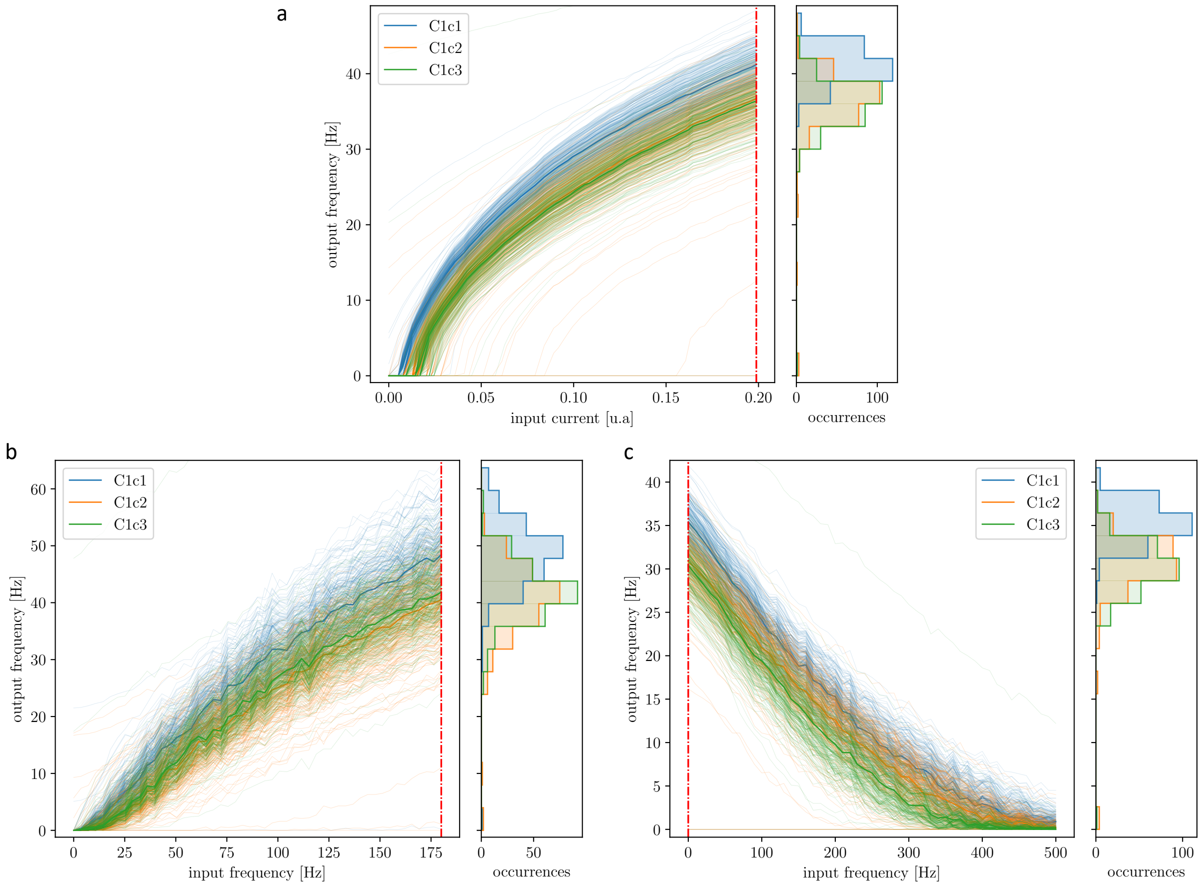

Supplementary Fig. S13: FI and FF curves of the neurons in three different cores of the DYNAP-SE chip, when subjected to: (**a**) common input current; (**b**) common excitatory poissonian spike trains (`AMPA` synapse) and (**c**) common inhibitory poissonian spike trains (`GABA_A` synapse). In each panel, the subplot on the right shows the histograms of the neurons' output frequency for the highlighted value of input current/frequency, separately for each core.

neuron (SIM-act) employing the $\delta-$rule (see Supplementary Fig. S14):

$$\mathrm{d}w_{syn} = -\lambda_w \frac{\partial E}{\partial w_{syn}} \propto -\lambda_w \sum_{(s,\mathtt{n})} (\text{DYN-act}(s,\mathtt{n}) - \text{SIM-act}(s,\mathtt{n}))\, H(\text{SIM-act}(s,\mathtt{n})) \tag{s11}$$

$$\mathrm{d}I_0 = -\lambda_c \frac{\partial E}{\partial I_0} \propto -\lambda_c \sum_{(s,\mathtt{n})} (\text{DYN-act}(s,\mathtt{n}) - \text{SIM-act}(s,\mathtt{n}))\, H(\text{SIM-act}(s,\mathtt{n})) \tag{s12}$$

where $H(x)$ is the Heaviside step function.

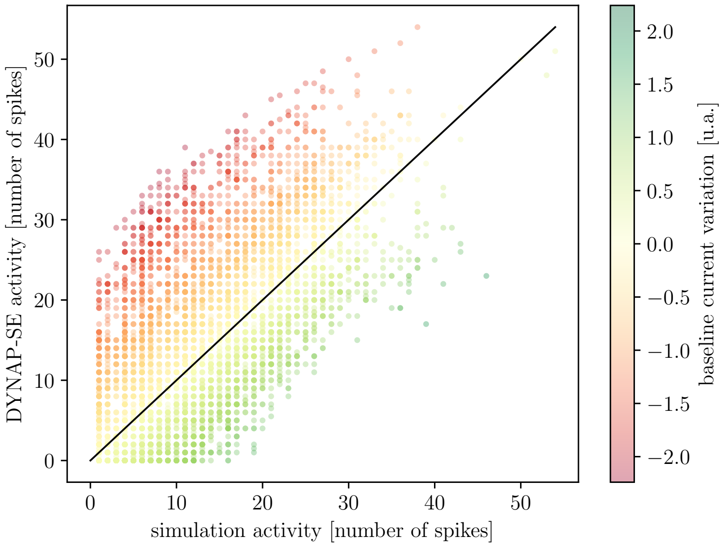

Supplementary Fig. S14: Scatter plot of the median DYNAP-SE neuronal activities as a function of the activity of simulated neurons when subjected to the same input stimuli. For each dot, the color indicates the related update on the value of the baseline input current shared among hardware neurons.

3.6.3 Optimized neuron association

To tackle the heterogeneity of analog neurons in the neuromorphic processor (see Supplementary Fig. S13), we optimized the association procedure between simulated and hardware neurons. To do so, for each output neuron $\mathtt{n}$, we considered the pairs $\{(s,\mathtt{n})\}_s$ containing the neuron $\mathtt{n}$, and analysed the activities of all the analog neurons j when different stimuli s were presented. These neurons were then ordered according to their capability in reproducing the response of neuron $\mathtt{n}$ in the simulations. To quantify this, we employed the χ^2 measure computed between the activities of the hardware and simulated neurons:

$$\chi_j^2 = \sum_s \frac{(\text{DYN-neur}_j(s,\mathtt{n}) - \text{SIM-act}(s,\mathtt{n}))^2}{\sigma_s^2} \tag{s13}$$

where $\text{DYN-neur}_j(s,\mathtt{n})$ is the activity of the hardware neuron j in the neuromorphic processor and σ_s has been heuristically fixed to the square root of the simulated activity. Each neuron $\mathtt{n}$ was then associated to the available (i.e., not already employed) DYNAP-SE neuron with lower χ^2 (see Supplementary Fig. S15).

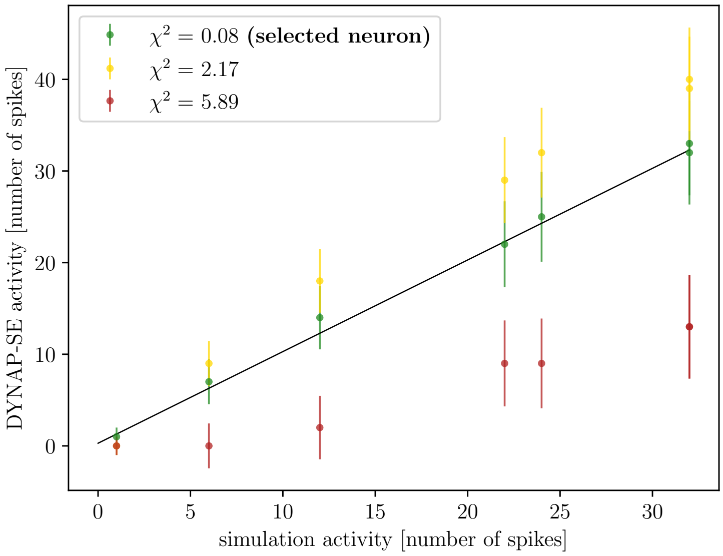

Supplementary Fig. S15: Activity of three different DYNAP-SE neurons as a function of the activity of simulated neurons when subjected to the same input. Note that hardware neurons associated with lower values of χ^2 (see legend) reproduce the activities of simulated neurons with higher accuracy. Error bars have been heuristically fixed to the square root of the simulated activity.

3.7 Summary of the employed parameters

	Quantity	SF-DIR	SC-BIO	First occurr. eq.
general	dt (integ. step)	1 ms	1 ms	(4)
neuron model	C_m	40 pF	40 pF	(4)
	g_L	2 nS	2 nS	(4)
	τ_{ex}	8 ms	8 ms	(4)
	τ_{in}	4 ms	4 ms	(4)
	E_L	−70 mV	−70 mV	(4)
	V_{th}	−50 mV	−50 mV	(4)
	t_{ref}	2 ms	2 ms	(4)
	w_{ext}	2 pA	2 pA	(5)
	ν_{ext}	1 kHz	1 kHz	(5)
architecture	$N^{(\text{input})} = 2n_{\text{FBG}}$	42	42	-
	$N^{(\text{intern})} = n_{intern}$	-	$6 \rightarrow 24$ (N)	-
	$N^{(\text{output})}$	$12^2 \rightarrow 32^2$ (N)	$12^2 \rightarrow 32^2$ (N)	-
pre-processing	LF	11	11	(7)
training	`W`	500 ms	500 ms	(13)
	`tS`	100 ms	100 ms	(13)
	m ($\mathrm{d}F/\mathrm{d}I$)	0.002 kHz pA^{-1}	0.002 kHz pA^{-1}	(13)
	`ind_per_batch`	10	10	(13)
	σ	5 mm	5 mm	(12)
	λ_m	$0.0125 \rightarrow 0.023$ (N)	$0.05 \rightarrow 0.16$ (N)	(15)
	λ_M	$1 \rightarrow 1.48$ (N)	$0.6 \rightarrow 1.9$ (N)	(15)
	δE_m	0.07	0.07	(15)
	δE_M	2.8	2.8	(15)
	h_{leak}	1.0	0.2	(17)
	I_{MAX} (input)	$14 \rightarrow 20$ (EE)	$14 \rightarrow 20$ (EE)	(18)
	I_{MAX} (intern)	-	16	(18)
	I_{MAX} (output)	16	16	(18)
	$\kappa_{decay,0}$ (input)	0	$5 \cdot 10^{-5}$	(19)
	$\mathtt{M}_{decay}$ (input)	0	0	(19)
	$\kappa_{decay,0}$ (intern)	-	$5 \cdot 10^{-5}$	(19)
	$\mathtt{M}_{decay}$ (intern)	-	$0 \rightarrow 1200$ (EE)	(19)
	$\mathtt{s}_{decay}$ (intern)	-	0.002 kHz	(19)
	$\kappa_{decay,0}$ (output)	0	$5 \cdot 10^{-5}$	(19)
	$\mathtt{M}_{decay}$ (output)	0	0	(19)
	$n_{\mathtt{bit}}$	$2 \rightarrow 7$ (Q)	$2 \rightarrow 7$ (Q)	(s10)
post-processing	$\tau_{\mathtt{out}}$	100 ms	100 ms	(8)
	$\alpha_{\mathtt{out}}$	0.9975	0.9975	(10)
DYNAP-SE	λ_w	-	$0.75 \cdot 10^{-6}$	(s11)
	λ_c	-	$2 \cdot 10^{-8}$	(s12)

Supplementary Tab. S5: Summary of the employed values for the main parameters of the two considered architectures. Some values have been modulated while analysing different degrees of Energy Efficiency (EE), Quantization (Q) or Number of output neurons (N).

4 Supplementary Results

4.1 Scalability to larger robotic surfaces and wiring complexity

The proposed solution is inherently scalable due to the modular design of the e-skin. Larger robotic surfaces can be covered by assembling multiple independent skin patches, each functioning autonomously. These patches can be processed in a distributed fashion, allowing parallel data acquisition and processing. Additionally, the design of each skin patch is highly flexible. Thanks to the nature of the fiber Bragg grating (FBG) technology, sensors can be embedded into patches of various shapes and dimensions, allowing for customized coverage tailored to specific surface geometries. Each optical fiber can host dozens to hundreds of FBG sensors, which significantly reduces wiring complexity while maintaining high spatial resolution. This flexible integration makes the approach suitable for both localized sensing and full-body tactile coverage.

To characterize scalability under replicated hardware conditions, we fabricated a second version of the e-skin, qualitatively replicating the design of the patch used in our main experiments. We then collected a new dataset by applying the same experimental protocol used in the original study. We tested the localization performance of the DYNAP-SE-compatible network using this new dataset, both with and without retraining on the new skin. The network achieved a median localization error of 5.25 mm with retraining and 5.49 mm without retraining. This represents a 4.5% increase in error without retraining, demonstrating a good degree of robustness and generalizability of our model across different skin instances. It is also worth noting that this performance drop likely represents an upper bound: both e-skins were manually fabricated, introducing minor structural differences. In real-world applications, industrial manufacturing processes would minimize such variability, likely reducing performance degradation. Further, FBG-based sensors are intrinsically robust and immune to electromagnetic interference, which is advantageous in noisy or harsh environments. Since multiple sensors can be multiplexed along a single optical fiber, wiring is greatly simplified compared to conventional electronic sensing solutions.

Supplementary References

[1] Luca Massari, Giulia Fransvea, Jessica D'Abbraccio, Mariangela Filosa, Giuseppe Terruso, Andrea Aliperta, Giacomo D'Alesio, Martina Zaltieri, Emiliano Schena, Eduardo Palermo, et al. Functional mimicry of ruffini receptors with fibre bragg gratings and deep neural networks enables a bio-inspired large-area tactile-sensitive skin. *Nature Machine Intelligence*, 4(5):425–435, 2022.

[2] Paul J Werbos. Backpropagation through time: what it does and how to do it. *Proceedings of the IEEE*, 78(10):1550–1560, 2002.

[3] Yujie Wu, Lei Deng, Guoqi Li, Jun Zhu, and Luping Shi. Spatio-temporal backpropagation for training high-performance spiking neural networks. *Frontiers in neuroscience*, 12:331, 2018.

[4] Emre O Neftci, Hesham Mostafa, and Friedemann Zenke. Surrogate gradient learning in spiking neural networks: Bringing the power of gradient-based optimization to spiking neural networks. *IEEE Signal Processing Magazine*, 36(6):51–63, 2019.

[5] Jason K Eshraghian, Max Ward, Emre O Neftci, Xinxin Wang, Gregor Lenz, Girish Dwivedi, Mohammed Bennamoun, Doo Seok Jeong, and Wei D Lu. Training spiking neural networks using lessons from deep learning. *Proceedings of the IEEE*, 111(9):1016–1054, 2023.

[6] Sumit B Shrestha and Garrick Orchard. Slayer: Spike layer error reassignment in time. *Advances in neural information processing systems*, 31, 2018.

[7] Ilyass Hammouamri, Ismail Khalfaoui-Hassani, and Timothée Masquelier. Learning delays in spiking neural networks using dilated convolutions with learnable spacings. *arXiv preprint arXiv:2306.17670*, 2023.

[8] Natalia Caporale and Yang Dan. Spike timing–dependent plasticity: a hebbian learning rule. *Annu. Rev. Neurosci.*, 31:25–46, 2008.

[9] P Jedlicka. Synaptic plasticity, metaplasticity and bcm theory. *Bratislavské lekárske listy*, 103(4/5):137–143, 2002.

[10] Francis Crick. The recent excitement about neural networks. *Nature*, 337(6203):129–132, 1989.

[11] Timothy P Lillicrap, Daniel Cownden, Douglas B Tweed, and Colin J Akerman. Random synaptic feedback weights support error backpropagation for deep learning. *Nature communications*, 7(1):13276, 2016.

[12] Timothy P Lillicrap, Adam Santoro, Luke Marris, Colin J Akerman, and Geoffrey Hinton. Backpropagation and the brain. *Nature Reviews Neuroscience*, 21(6):335–346, 2020.

[13] Dmitrii Zendrikov, Sergio Solinas, and Giacomo Indiveri. Brain-inspired methods for achieving robust computation in heterogeneous mixed-signal neuromorphic processing systems. *Neuromorphic Computing and Engineering*, 3(3):034002, 2023.
