# Supplementary material for: Bioinspired spiking architecture enables energy constrained touch encoding
Source: Nat Commun. 2026 Jan 28;17:2108. doi: 10.1038/s41467-026-68858-7 (PMC12953599; doi:10.1038/s41467-026-68858-7)
Supplement: Supplementary file 2 — Description of Additional Supplementary Information [file 41467_2026_68858_MOESM2_ESM.pdf]

Description of Additional Supplementary Files

File Name: Supplementary Movie 1
Description: localization performance of the SFDIR architecture trained under unconstrained conditions. (Left) Raster plot of the activity of SA1-mimicking neurons (green) and SA2-mimicking neurons (yellow) in the input layer, and of the activity of output neurons highlighted on the right panel. (Right) Animated scatter plot of the activities of the output neurons; the red and yellow markers indicate the actual and estimated points of contact, respectively. Horizontal bars display the instantaneous mean activity of the output layer and the instantaneous localization error. Stimulus detection and localization are triggered when the mean activity of the output layer consistently exceeds 0.02 Hz (see Methods).

File Name: Supplementary Movie 2
Description: localization performance of the SCBIO architecture trained under constrained energetic conditions. (Left) Raster plot of the activity of SA1- mimicking neurons (green) and SA2-mimicking neurons (yellow) in the input layer, and of the activity of output neurons highlighted on the right panel. (Right) Animated scatter plot of the activities of the output neurons; the red and yellow markers indicate the actual and estimated points of contact, respectively. Horizontal bars display the instantaneous mean activity of the output layer and the instantaneous localization error. Stimulus detection and localization are triggered when the mean activity of the output layer consistently exceeds 0.02 Hz (see Methods).

File Name: Supplementary Movie 3
Description: localization performance of the SCBIO architecture implemented on the DYNAPSE chip. (Left) Raster plot of the activity of SA1- mimicking neurons (green) and SA2-mimicking neurons (yellow) in the input layer, and of the activity of output neurons highlighted on the right panel. (Right) Animated scatter plot of the instantaneous activity of the output neurons; the red and yellow markers indicate the actual and estimated points of contact, respectively. Horizontal bars display the instantaneous mean activity of the output layer and the instantaneous localization error. After each indentation, a comparison between the filtered (left panel) and raw activities (right panel) is displayed (see Methods).
