# Supplementary material for: Bioinspired spiking architecture enables energy constrained touch encoding
Source: Nat Commun. 2026 Jan 28;17:2108. doi: 10.1038/s41467-026-68858-7 (PMC12953599; doi:10.1038/s41467-026-68858-7)
Supplement: Supplementary file 7 — Transparent Peer Review file [file 41467_2026_68858_MOESM7_ESM.pdf]

nature portfolio

Peer Review File

Bioinspired spiking architecture enables energy constrained touch encoding

Corresponding Author: Professor Calogero Oddo

This manuscript has been previously reviewed at another journal. This document only contains information relating to versions considered at Nature Communications.

Version 0:

Reviewer comments:

Reviewer #3

(Remarks to the Author)
I feel the authors have addressed my previous comments. Now the paper is suitable for publication at Nature Communications.

(Remarks on code availability)

Open Access This Peer Review File is licensed under a Creative Commons Attribution 4.0 International License, which permits use, sharing, adaptation, distribution and reproduction in any medium or format, as long as you give appropriate credit to the original author(s) and the source, provide a link to the Creative Commons license, and indicate if changes were made.
In cases where reviewers are anonymous, credit should be given to 'Anonymous Referee' and the source.
The images or other third party material in this Peer Review File are included in the article's Creative Commons license, unless indicated otherwise in a credit line to the material. If material is not included in the article's Creative Commons

license and your intended use is not permitted by statutory regulation or exceeds the permitted use, you will need to obtain permission directly from the copyright holder.
To view a copy of this license, visit https://creativecommons.org/licenses/by/4.0/
